# Union Instability and Fertility: An International Perspective

**DOI:** 10.1007/s10680-023-09668-1

**Published:** 2023-07-20

**Authors:** Ana Fostik, Mariana Fernández Soto, Fernando Ruiz-Vallejo, Daniel Ciganda

**Affiliations:** 1 Statistics Canada, Ottawa, Canada; 2 Programa de Población, FCS-UDELAR, Montevideo, Uruguay; 3 Facultad de Medicina, Universidad Nacional de Colombia, Bogotá, Colombia; 4 Max Planck Institute for Demographic Research, Rostock, Germany; 5 Instituto de Estadística, UDELAR, Montevideo, Uruguay

**Keywords:** Union instability, Cumulated fertility, Repartnering, Conjugal trajectory

## Abstract

In this article, we analyse the relationship between union instability and cumulated fertility among ever-partnered women in several regions across Europe and the Americas with different patterns of demographic behaviour in terms of fertility levels, union instability and fertility across partnerships. We hypothesise that the relationship between union dissolution and fertility might be less negative in contexts where repartnering is more prevalent. The analysis is performed on a large dataset of 25 countries, combining information from the Harmonised Histories of the Generation and Gender Programme with our own harmonisation of survey data from three Latin American countries. This allows for the inclusion of countries with differing prevalence of union instability as measured by (a) the proportion of women who separated by age 40, and (b) the proportion who repartnered by age 40. We first examine the prevalence of separation and repartnering during reproductive ages across regions, and we estimate the proportion of cumulated fertility attributable to unions of different ranks using a decomposition method. We then analyse the links between union instability and the number of children born by age 40 among ever-partnered and ever-repartnered women, using Poisson regression. Despite observing a high degree of heterogeneity in the proportions of births occurring in the context of repartnering both within and between regions, we find a pattern where a greater prevalence of repartnering by age 40 is accompanied by higher cumulated fertility in second or subsequent unions. Our multivariate findings reveal a negative statistical relationship between separation and cumulated fertility that is partially offset by repartnering in some contexts, and that the time spent in a union during the reproductive lifespan is a key determinant of cumulated fertility, regardless of national context and independently from age at union formation and union rank.

## Introduction

In a context of increasing separation and divorce rates, the relationship between union dissolution, repartnering and fertility outcomes becomes a central piece in the analysis of demographic dynamics and the projection of future fertility trends. However, analysing the effect of separation and repartnering on achieved fertility is a challenging task, mainly because the aggregate observed effects are the result of counteracting mechanisms. On the one hand, unstable partnership trajectories can result in fewer children through the consequent reduction of the time spent in union. On the other hand, the formation of second or higher order unions can result in larger family sizes than would have been achieved in the absence of interruptions if sharing biological children is seen as a way to consolidate new partnerships. In other words, whether the association between union instability and fertility is negative or positive depends, among other things, on whether additional birth(s) associated with repartnering outweigh the reduction of time spent in union caused by separation.

The links between union instability and fertility could be different across several European regions, North and Latin America, since these regions have historically differed in terms of the timing of the fertility transition –resulting in differing current fertility levels and timing of births– and have also experienced the changes associated with the second demographic transition at different paces and starting points.

The demographic literature has been concerned with the links between union instability and fertility since the early 1970s in the context of an increasing deinstitutionalisation of conjugal life, rising divorce rates and the displacement of marriage by cohabitation. The evidence emerging from a large number of studies conducted until now in Europe and North America is unambiguous: union dissolution, in the absence of repartnering, is negatively associated with the number of children by the end of the reproductive trajectory. Even though repartnering may help to partially compensate for this loss, the completed fertility of women who experience repartnering tends to be lower than the completed fertility of women that reach the end of their reproductive trajectories without experiencing separation or divorce. These results seem to be independent of the geographical context or time period (Beaujouan, 2010; Beaujouan & Solaz, 2008; Bélanger et al., 2010; Clarke et al., 1993; Kreyenfeld & Heintz-Martin, 2015; Meggiolaro & Ongaro, 2010; Pinnelli et al., 2002; Thomson et al., 2012; van Bavel et al., 2012; Winkler-Dworak et al., 2017).

Evidence for Latin America and the Caribbean is less abundant, and more ambiguous. Early studies, conducted in a period when the first demographic transition was still underway, in the 1960s and 1970s, found a consistently positive relationship between repartnering and fertility, especially among less educated women (Downing & Yaukey, 1979; Ebanks et al., 1974). More recently, analyses of Brazilian data have shown that the cumulated fertility of repartnered women was higher than the fertility of women who had remained in their first union, thanks to the contribution of births in second or higher order unions (Leone, 2002; Leone & Hinde, 2007). On the other hand, Fernández Soto (2019) found a negative effect of repartnering on the completed fertility of Uruguayan women compared to those who have not dissolved their first union, but also indications that this negative association is weakening among more recent cohorts.

Our main goal is to study the relationship between union instability and achieved fertility during the prime childbearing years, among ever-partnered women in a large number of Western countries. We study this relationship in multiple ways. First, exploiting the high variability of union dissolution and repartnering levels in our sample, we decompose cumulated fertility by conjugal status and then observe whether fertility outcomes at the aggregate level are associated with different levels of repartnering across societies. Secondly, moving to the individual level, we focus on the relationship between achieved fertility and the events of the conjugal trajectory of ever-partnered women during reproductive ages, examining whether the experiences of union dissolution and repartnering are associated with the number of children born up to age 40. Given the previously documented geographic variations in family demographic behaviour, throughout the analysis we focus on the potential regional patterns of variation in the relationship between union instability and fertility. Moreover, taking advantage of the unique harmonised dataset we created, we seek to explore whether the micro-level associations between union instability and fertility may be different in some Latin American countries than in several European regions and North America, where the first and second demographic transitions happened at differing starting points and paces, thus exhibiting varied fertility and union instability levels.

## Background

### Mechanisms in the Relationship Between Union Instability and Fertility

As mentioned earlier, one of the mechanisms linking union instability and fertility is the time spent in union. Thomson et al. (2012) have argued that any period of the reproductive life spent outside of a union is potentially detrimental to women’s fertility: the longer the periods of singleness during the fertile years, the more fertility is reduced. These periods can be the result of both delayed union formation, and of experiencing separation during the childbearing years. The loss of exposure to childbearing comes to an end when women repartner during reproductive ages; separated or divorced women who did not achieve their desired family size in their first union may try to achieve it in a new union (Kalmijn & Gelissen, 2007).

However, an important caveat of the possibility that individuals may accelerate childbearing in order to “catch-up” in a higher order union is that it depends on the timing of separation and repartnering: fecundability decreases with age and the window of opportunity to “catch-up” may close before individuals, particularly women, have a chance to fulfil their fertility intentions. Women’s age at repartnering is thus a key determinant of fertility in second unions, as has been shown for France (Beaujouan, 2011; Beaujouan & Solaz, 2013). In the same line, evidence for several European countries has shown that the earlier in the life course the separation, and the shorter the duration of the dissolved union, the more likely individuals are to become parents in new unions (Spijker et al., 2012). Moreover, couples may actually accelerate childbearing in higher order unions if they anticipate age-induced sterility. Evidence of this has been found among childless women in France (Beaujouan & Solaz, 2008) and for couples in the Netherlands (Kalmijn & Gelissen, 2007).

In societies where first union formation and first births are postponed, women who experience a separation might simply not have enough time to find a suitable new partner with whom to resume childbearing before they reach the end of their reproductive years. Thus, not only age at repartnering, but also age at first union formation, age at first birth and age at separation all have a potential effect on fertility after the demise of a union. As a result, the shorter the interval of time between separation and repartnering, the greater the odds that higher order unions might be fertile (Beaujouan, 2010, 2011).

Until now, we have discussed the total time spent in union as a mechanism through which union instability can reduce fertility, but there is at least one alternative mechanism that involves time in unions and can result in a positive relationship between repartnering and completed fertility. Thomson (2004) and Thomson et al. (2002) explain that there are at least three possible motivations for having children in second or higher order unions: the “commitment effect”, that is, having a child with a new partner as a way to consolidate the commitment to the new union; the “sibling effect”, where the motivation resides in giving a sibling to an existing child; and the "parenting status effect", where the motivation is to become a first-time parent among individuals who were still childless by the end of their previous union (Thomson, 2004; Thomson et al., 2002).

The studies reviewed until now demonstrate that the influence of union dissolution on reproductive outcomes is well established, but that reproductive outcomes may also influence (re)partnering behaviour. Several studies show that the presence, number and age of children from previous unions are also important determinants of childbearing in a new partnership after first union breakup (Beaujouan & Solaz, 2008; Griffith et al., 1985). Some of the early studies on fertility in second or higher order unions suggested that the number of previous children actually did not matter, as women would have at least a new birth in the higher order union to signal their commitment to their new partner, regardless of the number of previous children each partner had (Griffith et al., 1985). Empirical evidence for the “commitment value” of children was indeed found in several countries and periods (Griffith et al., 1985; Jefferies et al., 2000; Thomson et al., 2012). However, other findings suggest that parity at the time of repartnering does have an effect on fertility in higher order unions and, more specifically, that men and women are more likely to have children in the new partnership when at least one of the partners is childless when entering the union (Beaujouan, 2011; Beaujouan & Solaz, 2013; Buber & Prskawetz, 2000; Guzzo, 2014; Holland & Thomson, 2011; Spijker et al., 2012; Wineberg, 1988, 1990). When there are children from previous unions, the odds of a birth in the new couple increase as age of the youngest child at repartnering decreases (Beaujouan & Solaz, 2008; Buber & Prskawetz, 2000; Jefferies et al., 2000; Kalmijn & Gelissen, 2007).

Besides the effects on successive partnerships, the experience of a birth can reduce the odds of dissolution in the current union by increasing the costs of separation and providing a set of shared objectives. This has been shown to be true for the case of Italy and Spain by Coppola and Di Cesare (2008), who also found that unobserved characteristics might affect the risk of both union dissolution and childbearing, reducing the former and increasing the latter for “family-oriented” individuals.

In this article, we set out to study the relationship between union stability during prime childbearing years and achieved fertility. In order to examine how cumulated fertility is associated with the experiences in the conjugal trajectory during key reproductive ages, we decompose the cumulated fertility of women by conjugal status at age 40. This allows for an examination of the proportion of cumulated fertility that is achieved in a first union rather than in a second or higher order union.

In order to further study the relationship between achieved fertility and instability in the conjugal trajectory, we examine the association between cumulated fertility and: a) the experience of union dissolution, in and of itself; b) the experience of union dissolution in the absence of repartnering, compared to the experience of a separation followed by repartnering during reproductive ages; c) the total time spent in a conjugal union during the reproductive lifespan, which is the result of the timing of the events of union formation, dissolution and repartnering.

### Regional Patterns of Union Instability and Fertility

Several demographic behaviours related to union instability and fertility exhibit geographical patterns across regions.

Completed cohort fertility is overall higher in the United States than in Europe (Sobotka, 2017). In the European context, completed fertility in most recent birth cohorts is the lowest in Southern Europe, and the highest in Western and Northern Europe, whereas Central and Eastern Europe fall in between those extremes (Sobotka & Berghammer, 2021), as does Canada (Sobotka, 2017). The three selected Latin American countries (Colombia, Mexico, Uruguay) exhibit Total Fertility Rates that are considered low in the context of the region (CEPAL, 2008, 2014). These countries have reached advanced stages of the fertility transition in a way that make some of their demographic indicators comparable with those of European and North American countries. Uruguay stands out as a country where the first demographic transition occurred earlier, with lower fertility levels attained as early as the 1980s (CEPAL, 2014).^1^

Although fertility postponement is characteristic of the fertility decline across regions (Sobotka, 2017), the pathways towards low fertility differ among European regions: whereas an increase in childlessness predominates in Southern Europe and German-speaking countries, only-child families are more predominant in Central and Eastern Europe (Beaujouan et al., 2023; Sobotka & Berghammer, 2021).

The timing of childbearing also varies by regional context; across Europe, age at first birth is lowest in Eastern Europe, followed by Central Europe, and it is the highest in Western-Northern and Southern Europe (Sobotka & Berghammer, 2021). In North America, the overall average age at motherhood has been increasing since the 2000s, although little change has been observed in some socio-ethnic groups, particularly among Hispanic and African-American women (Mathews & Hamilton, 2016).

In Latin America, despite cultural, economic and sociopolitical transformations, age at first birth has remained rather stable across birth cohorts, which has been attributed to persistently high levels of teenage fertility (Batyra, 2016; Neal et al., 2018). Moreover, cross-national variations in the age at first birth are closely related to cross-country differences in age at first union and educational attainment (Esteve Palós & Florez-Paredes, 2018).

Focusing on union instability, its prevalence is much higher in North America, particularly in the United States, than in Europe (Raley & Sweeney, 2020; Wagner, 2020). However, divorce rates have increased in most European countries in recent decades (Amato & James, 2010), although at different paces and starting in different time periods across countries (Wagner, 2020). Within the European region, Southern and Eastern European countries exhibit much lower union instability than those in the Western-Northern region (Wagner, 2020). Within Central and Eastern Europe, we observe cross-national heterogeneity in the prevalence of divorce, where Estonia, Lithuania, Hungary and Russia have historically had higher divorce rates than other countries in these regions (Härkönen et al., 2020). Trends in family formation in the Eastern Bloc were impacted by the economic collapse of the region in the late 1980s. With the transition to an open market regime, non-marital fertility increased substantially in Central and Eastern Europe, until the early 2000s, when it started a downward trend (Klüsener, 2015).

Some countries in the Latin American region experienced a rapid deinstitutionalization of conjugal life in the past three decades: non-marital cohabitation increased in some countries where it was not previously common, such as in Colombia and Uruguay (Esteve Palós et al., 2012). In this context, marital separation and divorce have also increased in the Latin American region (Laplante et al., 2018; Liu et al., 2017). Uruguay stands out in the region for its earlier signs of the second demographic transition, that have become abundant in recent years (Binstock & Cabella, 2011; Cabella, 2009; CEPAL, 2021).

The second demographic transition framework can also be used to characterise the normative family changes in Southern and Western-Northern Europe in the last half century, towards less marriage, childbearing outside of marital unions and increased gender egalitarianism. Eastern Europe, on the other hand, experienced a re-traditionalisation characterised by a return to marriage as the normative family structure, and increased traditionalism in family values. Thus, family behaviour in this context can be more appropriately analysed in the “patterns of disadvantage” framework. For instance, childbearing outside of marriage and cohabitation are linked to lower, rather than higher educational attainment (Sobotka & Berghammer, 2021).

Such a pattern is also observed in the United States (Monte, 2019) and in Canada, where for example multiple-partner fertility has been linked to lower educational attainment and childbearing outside of residential unions, rather than to childbearing across partnerships (Fostik & Le Bourdais, 2020). Multiple-partner fertility is twice as common in the United States as in Europe; in the former around a fifth of mothers have children across partnerships (Stykes & Guzzo, 2019; Thomson et al., 2020). Within Europe, multiple-partner fertility is higher in the Western-Northern European countries than in Eastern and Southern Europe (Thomson et al., 2020).

The geographic patterns of demographic behaviour in terms of union instability and fertility described in this section lead us to think that geographic patterns of variation in the relationship between the two demographic processes could also be expected. Thus, we set out to identify regional patterns by analysing the prevalence of union dissolution and repartnering in each country and region, as well as by examining the association between cumulated fertility in second or higher order unions and the prevalence of repartnering at the aggregate level.

We further hypothesise that the association between fertility and union instability in the conjugal trajectory (both in terms of union dissolution and repartnering) might be less pronounced in national contexts and regions where union dissolution and repartnering are more prevalent and socially accepted. Two mechanisms underlie this assumption. First, a higher prevalence of repartnering during childbearing years creates larger pools of individuals at risk of having children in new unions. Secondly, similarly to the literature on the relative risks of dissolution of marriages and cohabiting unions (Liefbroer & Dourleijn, 2006; Pelletier, 2016), we posit that childbearing in second or subsequent unions may be less stigmatized and more socially acceptable in contexts where union dissolution and repartnering are not exclusively observed among a small subgroup of innovators.

## Analytical Strategy

### Data and Methods

Our main objective is to gain insight into the relationship between union instability and fertility by exploring the connection between the experience of transitions in and out of co-residential unions (either marriage or cohabitation) and achieved fertility.

To do so, we harmonised partnership and fertility trajectories information from national surveys in Colombia, Uruguay and Mexico.^2^ We later combined the information from these three Latin American countries with the Harmonised Histories dataset from the Generations and Gender Programme (GGP),^3^ which contains information on European and North American countries.^4^ The resulting dataset contains comparable information for 25 countries, some of which are observed more than once, in different time periods.

We perform our analysis on a sample of women aged 40 or older at the time of the survey,^5^ who were ever in a conjugal union (cohabiting union or marriage), who started their first union before age 40, and whose union was either ongoing at age 40 or ended before age 40 through separation or divorce.^6^ From this point onwards, we refer to the subset of women who experienced separation or divorce as "separated".

In order to conduct our analysis, we used biographical information on the number of unions, age at union formation and age at separation or divorce for each union episode. This allowed reconstructing conjugal trajectories up to the age of 40. We classed ever-partnered women according to whether, by age 40, they were: still in their first union; separated from a first union (and not repartnered); or in a second or subsequent union (repartnered). A number of descriptive statistics for this data can be found in Table 1 in the Appendix.

Firstly, we present a descriptive section in which we analyse the geographic patterns of union instability in terms of the prevalence across regions of union dissolution and of repartnering in a second or subsequent union. In order to assess the links between conjugal transitions and fertility, we also perform a descriptive decomposition of cumulated fertility among ever-partnered and among ever-repartnered women by union rank of births. In this analysis, we estimate the number of children and the fraction of cumulated fertility that can be attributed to first unions, to second or subsequent unions, and to out of union births. The procedure for such decomposition entails identifying the conjugal status of the respondent at the time of each birth (in first union; in second or subsequent union; outside of union), using biographical information on union and births histories in a person-year dataset. Once each birth has been associated to a specific conjugal status, respondents are classed according to the different conjugal situations in which they had children. These conjugal situations are: in first union only; in first and in second or subsequent union; in second or subsequent union only; other^7^; no children. The last step involves the within-respondent addition of births in each conjugal status and computing the proportion of cumulated fertility corresponding to each conjugal status.^8^

We then model the number of children ever born up to age 40 as the outcome of different characteristics of the partnership history, using Poisson regression for each country and survey iteration. Since our goal is to measure the result of the experience and the timing of conjugal transitions in the life course, the central variable of interest indicates the conjugal status by age 40; the categories of this variable vary in each model so as to allow focusing on different aspects of the process of union dissolution.

In the first three models, we estimate cumulated fertility by age 40 among ever-partnered women.

In our first model estimates, the main independent variable indicates ever having experienced a separation by age 40, as opposed to remaining in the first union (reference category). In the second model, we explore whether the links between union dissolution and fertility are different depending on whether the separation was followed or not by repartnering during reproductive ages. Thus, the categories of the main independent variable indicate: (a) experiencing separation and not forming a new union by age 40, or, (b) experiencing repartnering by age 40 (compared to still being in a first union as the reference category). In a third model, we use the same main independent variable as in model 2, but we add a measure of the total time spent in any union by age 40, as a way of disentangling the effects of the events of separation and repartnering themselves from the reduction of the time spent in a union on the coefficients for completed fertility. Our analytical approach entails including the total time spent in union^9^ in the models, instead of age at each separate event of the conjugal trajectory –first union formation, separation and repartnering. This approach allows us to control for the timing of events like union formation and dissolution while retaining in the analysis both women who had stable conjugal trajectories and those who experienced separation, repartnering or both. This allows the key comparison with those still in their first union. In order to control whether the time spent in union happened at earlier or later ages of the reproductive life span, we add a covariate indicating whether first union formation occurred before or after age 25.

Finally, we estimate a fourth model for cumulated fertility by age 40, this time only among ever-repartnered women. Here, the central variables of interest are the time spent in a first union and the time spent in a second or subsequent union,^10^ in order to understand whether time spent in higher order unions has a distinctive effect on cumulated fertility. As a way to control whether the time spent in first and in second or higher order unions happened at earlier or later ages of the reproductive life span, we include in this model covariates indicating whether first union formation occurred before or after age 25, and whether repartnering occurred before or after age 30.

All our models control for age, type of first union (marriage or not) and whether the respondent has attained a higher educational attainment level (ISCED levels 5 or 6) at the time of the survey.

### Geographic Perspective

We incorporate a geographic perspective throughout the analysis as a way to a) examine whether countries that have been less explored in the previous literature stand out with respect to the link between union dissolution, repartnering and fertility, and; b) to understand whether the observed results are context dependent. To classify the countries into regions, we follow the classifications proposed by Zeman et al. (2018) and by Sobotka and Berghammer (2021) for the European countries, and we divide the countries of the Americas into Latin American and North American countries. The 25 countries in our sample are then classified in the following way:

1. *Southern Europe*: Italy and Spain;
2. *Central Europe*: Czech Republic, Estonia, Hungary, Lithuania and Poland;
3. *Eastern Europe*: Bulgaria, Romania Belarus, Georgia, and Russia;
4. *Western and Northern Europe*: Austria, Belgium, France, Germany, Netherlands, Norway, Sweden, and the United Kingdom;
5. *North America*: Canada and the United States;
6. *Latin America*: Colombia, Mexico, and Uruguay.

Our classification of European countries slightly differs from that of Zeman et al. (2018) and that of Sobotka and Berghammer (2021), in that we use broader regions for Eastern Europe and for Western and Northern Europe. This decision is based on the number of countries available in our sample, to avoid groups of countries containing only two observations. We highlight European subregional differences in our analysis, when appropriate, between the Southeastern countries (Bulgaria and Romania) and the Eastern region (Belarus, Russia and Georgia), as well as among the German-speaking countries (Austria and Germany), the Western countries (Belgium, France, the United Kingdom, and the Netherlands) and the Nordic countries (Norway and Sweden).

## Descriptive Findings

### Geographical Patterns of Union Instability

Across regions, ever-partnered women’s most common conjugal status by age 40 was to be in their first union. However, union instability levels, both defined as the percentage separated and the percentage repartnered by age 40, exhibited great variation between regions, and in some cases also within.

**Fig. 1:**
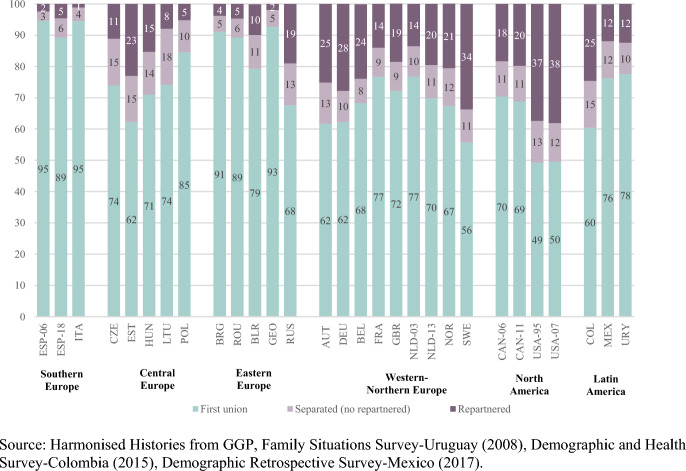
Distribution of ever-partnered women by conjugal status by age 40 (percentage). Ever-partnered women aged 40 and older at the time of the survey. By regions, country and year of survey

Overall, Southern and Eastern Europe stand out as the regions with the lowest prevalence of separated women by age 40, with Spaniards and Italians exhibiting the lowest shares of separated women (3% to 6%) of all observed countries (Fig. 1), as well as the highest mean ages at separation (see Table 1 in Annex). Some of the countries of the Eastern European region –Bulgaria, Georgia and Romania follow closely (5% to 6% of separated women). Russia exhibits an exceptionally high share of separated women (13%), as well as younger mean ages at separation, in the context of the Eastern European region.

A high degree of heterogeneity in the prevalence of separation by age 40 is observed among women in Western and Northern European countries, ranging from 8% of ever-partnered women in Belgium (2005) to 13% in Austria. In North and Latin America, this percentage oscillates between 10% (Uruguay) and 15% (Colombia), the second to highest proportion of separated women in the sample (Fig. 1). Mean age at separation is also lower in North and Latin America, albeit to a lower extent in Canada (2006) and Uruguay (see Table 1 in Annex).

Except for Poland, the Central European region exhibits the highest proportions of separated women outside of Latin America, ranging from 14% in Hungary to 18% in Lithuania, the highest share among the observed countries (Fig. 1).

Focusing now on the percentage of women who had repartnered in a second or subsequent union by age 40, again women in Southern and some Eastern European countries stand out by the very low prevalence of repartnering, particularly Spain (2006), Georgia and Italy (between 1% and 2%), followed by women in Bulgaria, Spain (2018) and Romania (between 4% and 5%). Again, repartnering is, however, much more common in Russia –where almost one in five ever-partnered women have repartnered by age 40 (19%)– than in the rest of Eastern Europe (Fig. 1).

The share of repartnered women in the Central European countries ranges between 5% in Poland and 15% in Hungary, with the exception of Estonia, where almost a quarter (23%) of ever-partnered women had repartnered by age 40 (Fig. 1).

The prevalence of repartnering in the Northern and Western European countries ranges from 14% in the Netherlands (2003) and France to more than a quarter of ever-partnered women in Belgium, the German-speaking countries (Austria and Germany) and Sweden (Fig. 1).

In North America, the prevalence of repartnering is much higher in the United States, where ever-partnered women exhibit the highest percentage of repartnering by age 40 in the entire sample: 37%-38% (1995 and 2007) (Fig. 1). Women in the United States also stand out for the youngest mean ages at repartnering (see Table 1 in Annex).

Women in Latin American countries tend to repartner less frequently than their Western-Northern European or their North American counterparts, with the notable exception of Colombia, where women are twice as likely as those in Mexico or Uruguay to be in their second or subsequent union by age 40 (25% in the former and 12% in the two latter) (Fig. 1).

In this section, we observed some geographical patterns in regards to the prevalence of union instability across regions. Women in Southern and Eastern Europe exhibit later ages at separation and the lowest levels of union dissolution and of repartnering by age 40, with the notable exception of Russia, previously documented in Härkönen et al. (2020). Central European and Colombian women exhibit the highest proportions experiencing separation during the reproductive years, while women in Sweden and the United States stand out for the highest shares of repartnering - and in the case of the United States, also for the earliest ages at repartnering.

### Cumulated Fertility Among Ever-Partnered Women: Descriptive Decomposition of Cumulated Fertility

Among ever-partnered women in our sample, cumulated fertility at age 40 is the lowest among women residing in the European regions, with most countries showing an average cumulated fertility of under two children per woman; it is the lowest for women in Spain (2018), at 1.4 children per woman.

Cumulated fertility is higher for North American women, those in Nordic countries (Norway and Sweden) and some Western European countries (France, United Kingdom) as well as in Poland and Georgia. The highest fertility levels in our sample are found in Latin American countries, particularly in Colombia and Mexico where the average number of children per woman is close to three (see Table 2 in Appendix). These two countries also stand out for having the lowest mean age at childbearing among the countries in our sample, around 22.5 years, while the mean age at childbearing among Uruguayan women is more than 2 years older, at 24.8 years (see Table 1 in Appendix).

**Fig. 2:**
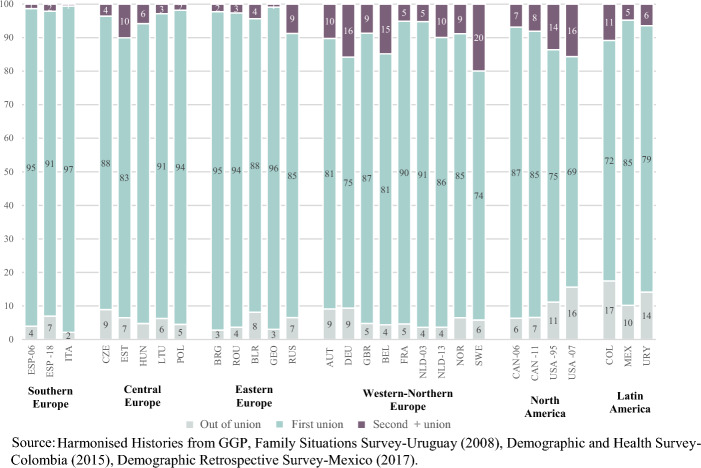
Share of cumulated fertility by conjugal status and rank of union of birth (percentage). Ever-partnered women aged 40 and older at the time of the survey. By regions, country and survey iteration

Figure 2 shows that, whereas in all countries and regions most cumulated fertility among ever-partnered women is attributable to childbearing in first unions, there is a high degree of variation in the cumulated fertility attributable to second or subsequent unions, which varies from less than 1% of cumulated fertility in Italy to 20 % of cumulated fertility in Sweden. The share of cumulated fertility attributable to births in second or subsequent unions is very low among ever-partnered women in Southern Europe, in all Eastern European countries but Russia, and in some Central European countries (Poland, Lithuania, the Czech Republic), representing 4% or fewer of all births among ever-partnered women (Fig. 2). This finding complements the portrait of the Southern and Eastern European regions as those where conjugal and childbearing trajectories during reproductive ages revolve the least around union instability, in terms of the experience of dissolution, repartnering or childbearing within a second or subsequent union, as shown in Sect. 2.2.

Among women residing in Northern and Western Europe, fertility in higher order unions oscillates from relatively lower shares in France and the Netherlands (2003), where about 5% of births are attributable to higher order unions, to somewhat higher shares in United Kingdom, Norway and the Netherlands (2013), and much higher shares in Belgium, Germany and Sweden, the country with the largest share of repartnered women by age 40 among the European countries; 20% of ever-partnered Swedish women’s cumulated fertility is attributable to births in higher order unions, the highest observed in any region (Fig. 2).

In North America, births in second or higher order unions represent between 7% to 8% births per woman in Canada (2006 and 2011) and 14% and 16% of births in the United States (1995 and 2007). A great degree of variability is also observed among women in the Latin American countries, where the share of cumulated fertility attributable to second or higher order unions in Colombia (11%) is about twice the share observed in Mexico and Uruguay –despite overall cumulated fertility being the highest among all observed countries both in Mexico and in Colombia (Fig. 2).

The Latin American countries, especially Colombia, also stand out regarding the share of out of union births, a finding that is consistent with previous research (Laplante et al., 2018). However, this is not exclusive to Latin American countries: despite Colombia being one of the countries with the highest share of cumulated fertility outside of union, this percentage is similarly high among women in the United States (2007) (Fig. 2).^11^

**Fig. 3:**
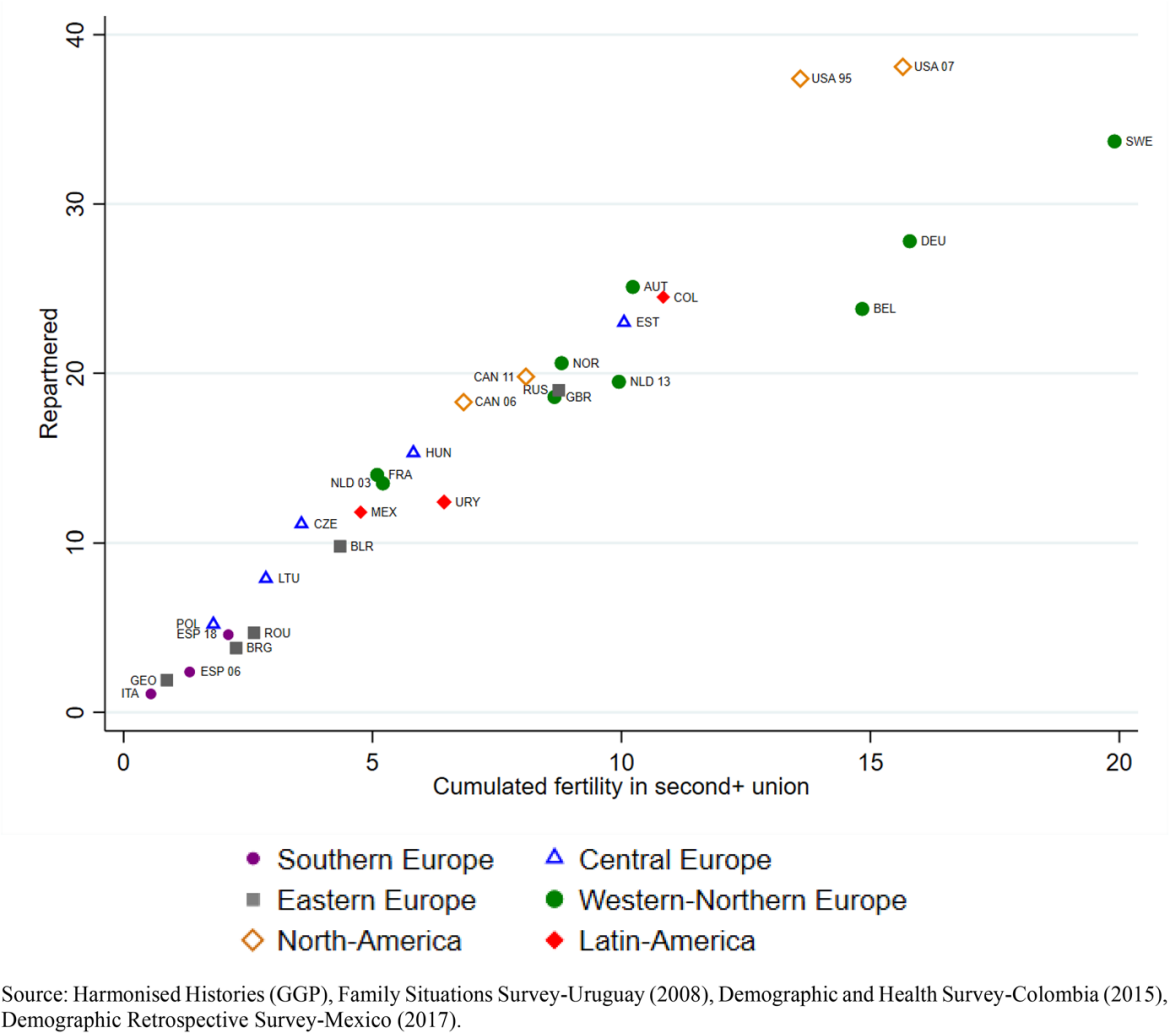
Cumulated fertility in second or subsequent unions and repartnered in second or subsequent unions (percentage). Ever-partnered women aged 40 and older at the time of the survey. By regions, country and survey iteration

Overall, we observe a pattern in which the share of cumulated fertility attributable to births in second or subsequent unions is higher in countries where the share of ever-partnered women who had repartnered by age 40 is also higher (Fig. 3). There is a geographical pattern in which the share of births attributable to repartnering is associated with the proportion of repartnered women. Very low prevalence of repartnering in Southern and most of Eastern Europe is associated with lower shares of cumulated fertility in such unions. We observe this pattern as well in countries such as Belarus and especially Russia, which have higher prevalence of women in second or subsequent unions than other Eastern countries, and also exhibit higher shares of fertility in such unions. Central European countries are more scattered in the prevalence of repartnering, but also show higher cumulated fertility at higher levels of repartnering, particularly in Estonia. Among the European countries, Sweden exhibits the highest percentages of both repartnering by age 40 and cumulated fertility in second or subsequent unions. This association is less marked in the United States, where the share of repartnered women is slightly higher than in Sweden, but the percentage of births attributable to second or subsequent unions is lower.

### Cumulated Fertility Among Ever-Repartnered Women: Descriptive Decomposition of Cumulated Fertility

**Fig. 4:**
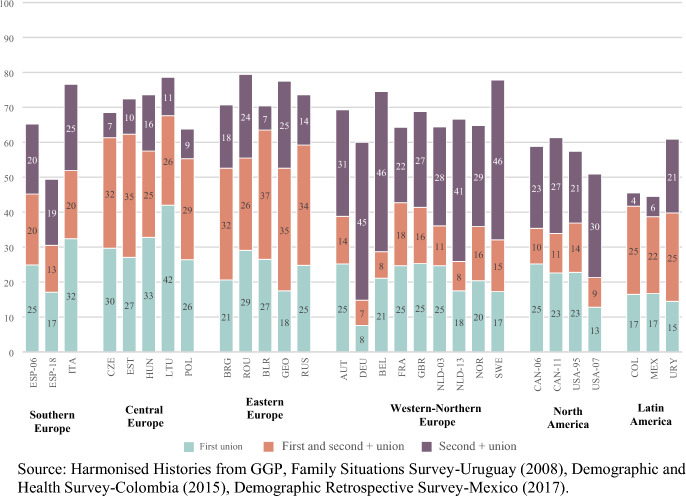
Share of repartnered women by childbearing trajectory according to births in first union, births in second or subsequent union, births in both first and second or subsequent union and no births (percentage). Ever-repartnered women aged 40 and older at the time of the survey. By regions, country and survey iteration*. *Note: The rest of the distribution corresponds mainly to births outside of union, and the combinations of this with first and second union. The category includes the following situations: all births outside of union; births in first union and outside of union; births in second or subsequent union and outside of union; births in first and second or subsequent union and outside of union situations, can be consulted in Table 2 of the appendix

Focusing now on women who had repartnered by age 40, Fig. 4 shows that a large fraction of these women had births in second or subsequent unions; either as a combination of having births both in first and in second or higher order unions, or by having births only in second and subsequent unions.^12^

The proportion of women with births only in second or subsequent union is the lowest in two of the Latin American countries (Colombia and Mexico), in the Central European countries and in Belarus. In the Southern European countries, this share is between one fifth and one quarter of all repartnered women. Repartnered women in Northern and Western Europe stand out, on the contrary, for having the highest shares of births only in second or subsequent unions; in countries like Belgium, Germany and Sweden, close to half the births to repartnered women happened in the context of higher order unions (Fig . 4).

The trajectories of repartnered women in Central and Eastern Europe are characterized by the experience of births both in their first and in their second or subsequent unions; between a quarter and a third of them had this type of childbearing trajectory by the time they were 40 years old. This type of trajectory is also more frequent among ever-repartnered Latin American women, a quarter of Colombians and Uruguayans having had children both in the first union and in second or subsequent unions. This share is slightly lower in Mexico (22%) (Fig. 4).

Among North American ever-repartnered women, having children only in the first union is about as common as having children only in second or subsequent unions, except for the United States (2007), where childbearing within the first union is less common than in second or subsequent unions (Fig. 4).^13^

The analysis of the share of births by conjugal setting focusing only on ever-repartnered women sheds light on the fact that childbearing within repartnering is relatively common in some regions where union instability is the least prevalent, such as in Southern Europe. We also observe that childbearing both in the first union and in subsequent unions is more prevalent in certain regions, particularly among repartnered women in Latin America, Central and Eastern Europe.

## Poisson Regression Models

**Fig. 5:**
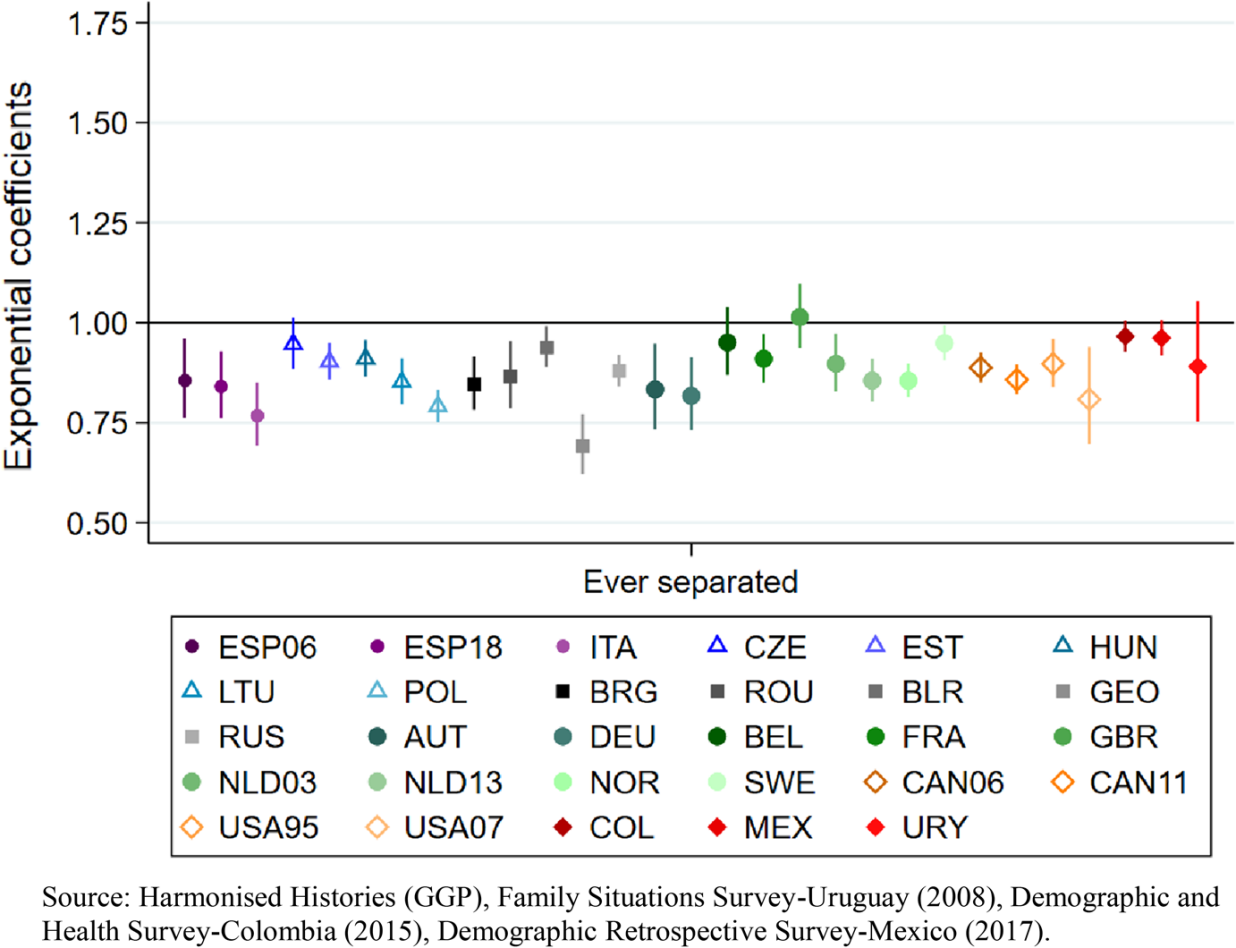
Model 1. Cumulated fertility (dependent variable) among ever-partnered women aged 40 and older at the time of the survey. Poisson regression coefficients (exponential) for experiencing separation by age 40 (reference category: in first union), controlling for first union before age 25, educational attainment, age and first union type. By regions, country and survey iteration

Our first model examines the overall impact of experiencing union dissolution on cumulated fertility by age 40 among ever-partnered women. As expected, in almost all regions and national contexts-periods we observe lower fertility among women who experienced separation by age 40, as denoted by the exponentiated coefficients below 1. Among women in Southern and Eastern Europe and in North America, having separated by age 40 is consistently associated with lower cumulated fertility. This is also the case for women in Central Europe, except for the Czech Republic, where the coefficient is negative but not statistically significant, as denoted by confidence intervals touching the horizontal black line (Fig. 5).^14^ In Western Europe, the coefficients are negative for the German-speaking and the Nordic countries, as well as for France and the Netherlands. In Belgium and in the United Kingdom, the coefficients are not statistically significant.

Latin America is the only region in which none of the countries show a significant negative coefficient for the relationship between the experience of union dissolution by age 40 and fertility.

Another relevant result for model 1 (see Table 3 in Appendix) is that women who formed their first union after age 25 tend to have lower fertility than women who formed their first union before that age. A result that is consistent across all contexts and time periods.

**Fig. 6:**
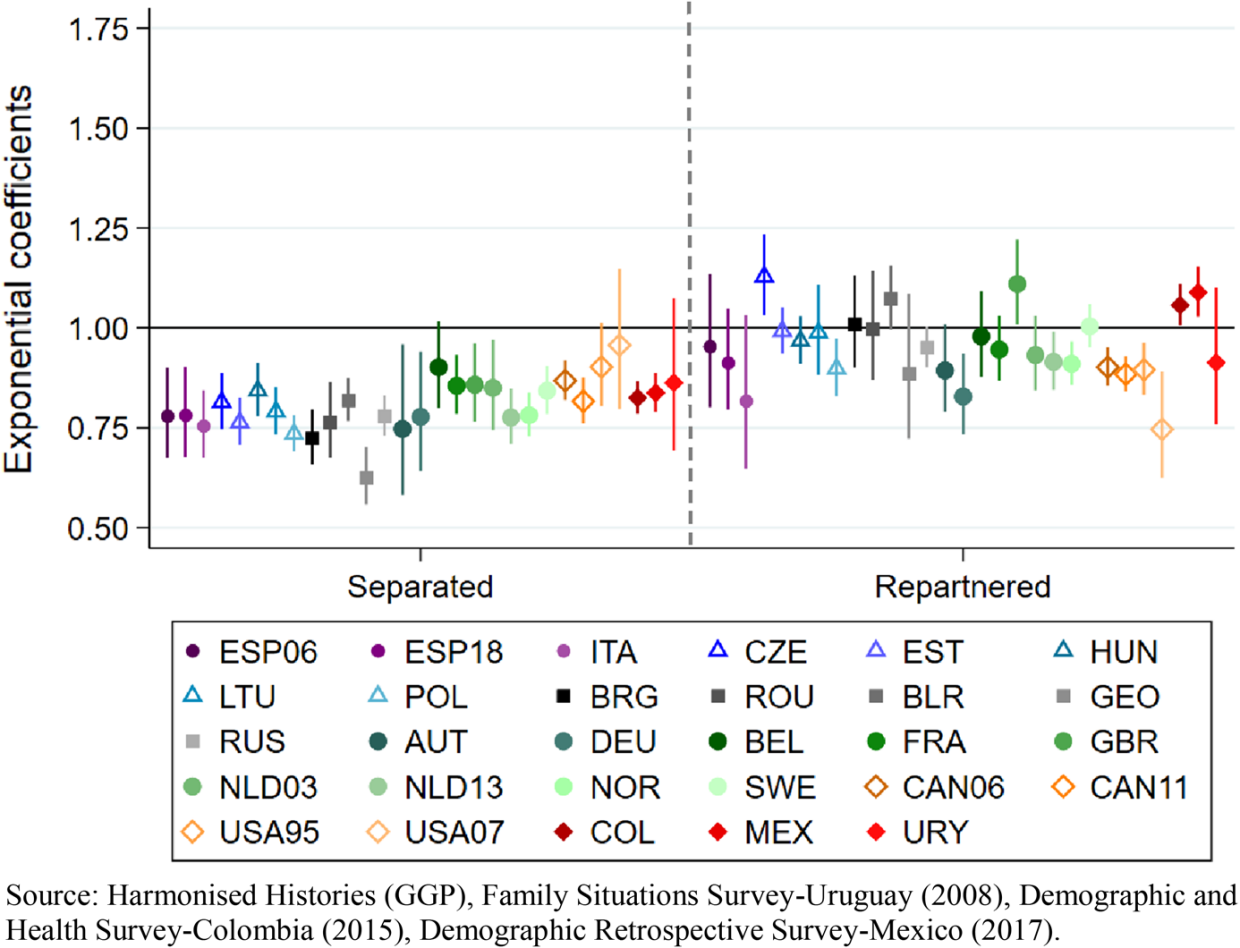
Model 2. Cumulated fertility (dependent variable) among ever-partnered women aged 40 and older at the time of the survey. Poisson regression coefficients (exponential) for experiencing separation by age 40 and for repartnering by age 40 (reference category: in first union), controlling for first union before age 25, educational attainment, age, and first union type. By regions, country and survey iteration

Our second model makes the distinction between separations that were followed by repartnering and those were women remained separated by age 40, thus allowing examining whether repartnering after a separation may help compensate for the detrimental effect of union dissolution on fertility (Fig. 6). The exponentiated coefficients for having separated without subsequently repartnering show a clear negative sign in most regions and countries, with the exception of a few cases where the effect is nonsignificant –Belgium in Western Europe, the United States in the North American region (1995 and 2007) and Uruguay in Latin America.

When we consider the trajectories of women who formed new unions after separation, there is a very clear indication that repartnering does help compensate for the fertility lost to union dissolution. Across regions, most coefficients are not statistically significant, indicating that the fertility of repartnered women in most countries is statistically indistinguishable from the fertility of women in intact unions (Fig. 6). Some coefficients for repartnering before age 40 are negative in several European countries (Poland, Germany, Netherlands 2013, and Norway), and across the North American countries, indicating that repartnering is associated with lower fertility than remaining in a first union by age 40. In most cases, however, the negative coefficients are lower for repartnering by age 40 than for experiencing separation without repartnering. Moreover, in four of the countries of our sample, repartnering is positively associated with fertility, as indicated by positive and significant coefficients. This is the case of the Czech Republic, the United Kingdom, Colombia and Mexico (Fig. 6).^15^

As in the previous model, having formed a first union after age 25 is consistently associated with lower fertility across all contexts and time periods (see Table 4 in Appendix).

**Fig. 7:**
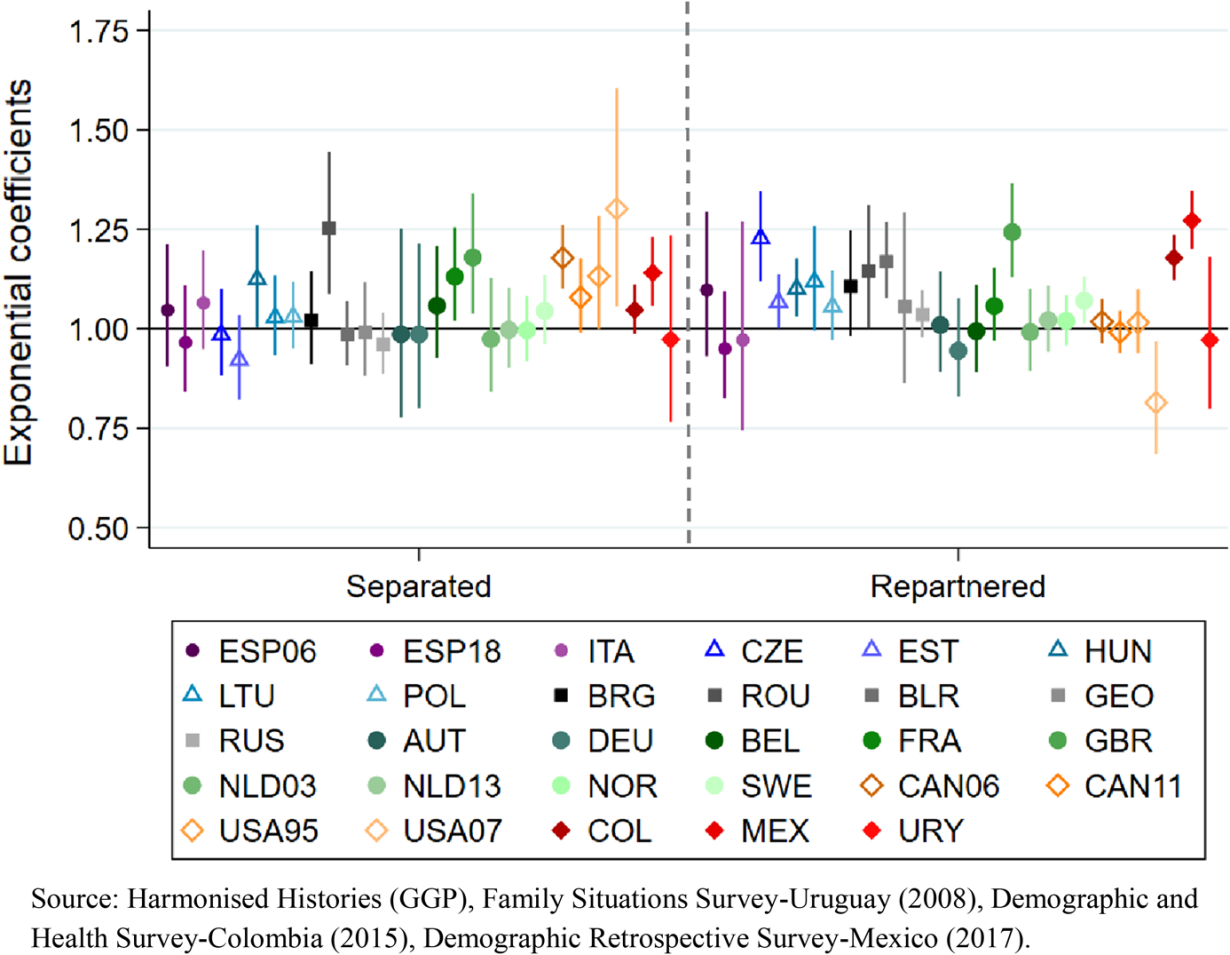
Model 3. Cumulated fertility (dependent variable) among ever-partnered women aged 40 and older at the time of the survey. Poisson regression coefficients (exponential) for experiencing separation by age 40 and for repartnering by age 40 (reference category: in first union), controlling for total time spent in union by age 40, first union before age 25, educational attainment, age, and first union type. By regions, country and survey iteration

Our third model adds the total time spent in union by age 40 to the previous estimation (Fig. 7). Without exception, all the negative coefficients observed for experiencing separation without repartnering in the previous model now become either non-statistically significant or positive. A positive association between being separated from the first conjugal union by age 40 and cumulated fertility is now observed among ever-partnered women in some Western European countries (France and the United Kingdom), Canada (2006) and the United States (2007) in North America, and Mexico in Latin America.^16^ In all observed countries from Southern, Central and Eastern Europe -except Hungary and Romania-, no significant association is now found between being separated and cumulated fertility by age 40.

The introduction of the control for total time in a union in model 3 entails a change by which the coefficients for repartnering that were negative in the previous model are now not statistically significant, with the only exception of the United States (2007). This means that, across regions, the relationship between being in a second or subsequent union and cumulated fertility by age 40 is now either positive or not statistically distinguishable from continuing in an intact first union by age 40 (Fig. 7).

In North America -with the above-noted exception-, no association is observed between repartnering and cumulated fertility when controlling for time in a union; the same is true across countries in Southern Europe and in Western Europe except for the United Kingdom and Sweden, where the coefficient is positive. In Central Europe, a positive association is observed between repartnering and fertility in the Czech Republic, Estonia, and Hungary. In Eastern Europe, such a positive association is observed in Romania and Belarus (Fig. 7).

Among women in the Latin American region, the coefficients for those residing in Colombia and Mexico also denote a positive association between the two variables (Fig. 7). Interestingly, the strongest positive association between repartnering and fertility is found in Mexico, which despite not standing out for a high prevalence of repartnering by age 40 does stand out as the country with the highest cumulated fertility in our sample. The fact that Uruguay, the third Latin American country in our sample, does not present similar results to Mexico and Colombia in regard to the link between repartnering and fertility is not surprising considering that Uruguay has distinct demographic characteristics to other countries in Latin America, at least in terms of the second demographic transition (Fernández Soto & Laplante, 2020; Nathan et al., 2016).

Once the time spent in a union is included as a covariate in model 3, the negative coefficient we had consistently observed across regions for having entered a first union after age 25 is now only observed in some contexts (see Table 5 in Appendix), particularly in Latin American and North American countries –except the United States (2007). In the Southern, Central, Western and Northern European countries, the negative association between later union formation and cumulated fertility disappears once the total time in union is accounted for, except for Sweden. Moreover, the association becomes positive in Italy and Germany. In Eastern Europe, the statistical relationship between the two variables mostly disappears, except for a persistent negative coefficient for Russia and a now-positive coefficient for Georgia.

**Fig. 8:**
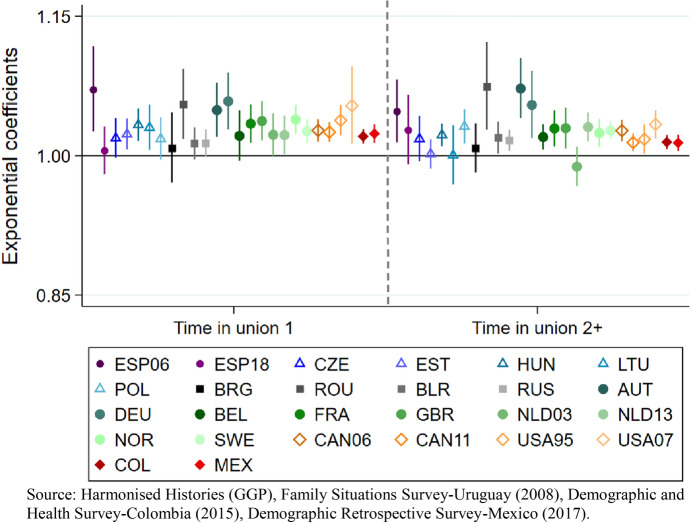
Model 4. Cumulated fertility (dependent variable) among ever-repartnered women aged 40 and older at the time of the survey. Poisson regression coefficients (exponential) for time spent in a first union and for time spent in a second or subsequent union, controlling for first union before age 25, repartnering before age 30, educational attainment, age, and first union type. By regions, country and survey iteration. Note: in the case of Georgia, Italy and Uruguay, the number of cases is not sufficiently representative to make statistical inferences

Our fourth model only includes ever-repartnered women and examines the time spent in a first and in a second or subsequent union on cumulated fertility as key predictive variables (Fig. 8).^17^

Across countries in North and Latin America, as well as in most Western-Northern European countries and in Spain (2006) the coefficients both for time spent in a first union and for time spent in second or subsequent unions are positive and significant. In Central and Eastern Europe, the coefficients for time spent in a second or subsequent union are positive and, in some cases, significant (Hungary, Poland, Romania, Belarus, Russia). Most coefficients for the first union in these two regions are not statistically significant despite being positive.

Model 4 introduced controls for the timing of the first union and also for the timing of the second or subsequent union formation (see Table 6 in Appendix). Among repartnered women, a negative coefficient of forming a first union after age 25 is observed in some countries. Having repartnered after age 30, entails a negative coefficient in Estonia, Canada (2011), the United States (1995), Colombia, and Mexico, while the coefficients for the rest of the countries are not statistically significant.

## Discussion

Our analysis revealed that union instability in prime childbearing years is rare among ever-partnered women in Southern and most of Eastern Europe (with the notable exception of Russia, previously highlighted by Harkonen et al. (2020), where conjugal and childbearing trajectories during reproductive ages revolve the least around dissolution and repartnering. This finding confirms, for ever-partnered women during childbearing ages, a previously documented pattern of overall lower prevalence of union dissolution in Southern and Eastern Europe (Wagner, 2020). These results are also in line with the more conservative family norms that are present in these European regions (Sobotka & Berghammer, 2021; Sobotka & Toulemon, 2008).

On the other hand, experiencing union dissolution was much more frequent for women in Central Europe (except Poland), in Western-Northern European countries, in North America, and in Colombia among the Latin American countries. However, a higher prevalence of union dissolution by age 40 is not necessarily met with higher levels of repartnering among Central European women (except among Estonians, and to a lesser degree, Romanians). Repartnering before age 40 is much more common in the German-speaking European countries (Austria and Germany), in Sweden, the United States and Colombia. Albeit at lower levels, second or subsequent union formation was also not rare in the other Western and Northern European countries, and in Russia.

Although most childbearing among ever-partnered women happens in first unions, there is a large degree of heterogeneity across national contexts in the proportion of births that take place in second or subsequent unions. One of our goals was to gain an understanding into whether a higher prevalence of repartnering during childbearing years was associated with fertility outcomes, across a wide range of cultural and socioeconomic contexts. The descriptive analysis revealed a pattern where the higher the share of repartnered women, the higher the proportion of cumulated fertility in second or subsequent unions. In this sense, women in regions such as Southern, Eastern and some of Central Europe repartner very infrequently (a finding in line with previous research by Gałęzewska et al. (2017), and the part of their cumulated fertility that is attributable to second or subsequent unions is negligible. On the other end, the United States and Sweden stand out for much more frequent repartnering during childbearing ages, and for a much larger fraction of fertility that is attributable to second or subsequent unions. The association is, however, nuanced in the United States, where the percentage of cumulated fertility in second or subsequent unions is more comparable to Western European countries where repartnering is less common.

The multivariate analysis allowed isolating some of the mechanisms that link instability in the conjugal trajectory to cumulated fertility (net of confounders such as, age at first union formation, first union type, educational attainment, and birth cohort), notably the roles played by (a) experiencing union dissolution before age 40; (b) experiencing repartnering before age 40; and (c) the total time spent in a conjugal union before age 40.

As expected, and in line with previous findings, experiencing union dissolution –rather than remaining in the first union during the prime childbearing years– is mostly associated with lower cumulated fertility. However, such a negative relationship was not found in the Czech Republic, Belgium, the United Kingdom, and the three Latin American countries, Uruguay, Mexico and Colombia.

Once we made the distinction between experiencing union dissolution and remaining single, compared to repartnering following the demise of the first union, we observed a compensating effect of forming a new union on cumulated fertility. In a few countries, the relationship between repartnering and cumulated fertility is even positive, meaning that repartnered women tend to have higher fertility than those in intact unions. This was the case in some of the same countries where the separation itself was not statistically associated with cumulated fertility (Czech Republic, the United Kingdom, Mexico and Colombia).

The total time spent in union before age 40, a key variable linked to the *timing* of union formation and dissolution, showed a positive association with cumulated fertility in most national contexts. Moreover, once this control was added to the models, we identified changes in the previously observed associations between union instability and fertility. Most notably, experiencing separation – and remaining single – no longer has an association with cumulated fertility in all countries, except in some where the association is now actually positive (France, Canada, and the United States). This suggests that the negative association between union dissolution and cumulated fertility is not only offset by repartnering, but also mediated by the time spent in union.

The association between repartnering and fertility is also strongly influenced by the amount of time spent in a conjugal union during the prime reproductive years: once this variable is controlled for, repartnering becomes either no longer associated with cumulated fertility, or positively associated. Among women in Southern Europe, the conjugal trajectory and cumulated fertility are not statistically linked once the time in first union is taken into consideration; this finding is consistent with their much higher ages at first union formation and also at repartnering, among the highest observed in the sample. On the other hand, the relationship between repartnering and fertility is clearly positive in the Czech Republic, Estonia and Lithuania in Central Europe, Romania and Belarus in Eastern Europe, the United Kingdom and Sweden in Western-Northern Europe, and Mexico and Colombia in Latin America.

These results shed light on the fact that union instability and repartnering in and of themselves might be neutral or even positive for fertility, as long as the *timing* of dissolution and of new union formation are not protracted during the prime reproductive ages.

The one exception to this overall pattern is found in the United States (2007), where repartnering is negatively associated with cumulated fertility. This result is puzzling given that women in the United States exhibit the highest share of multiple-partner fertility among industrialized nations (Thomson et al., 2020). This apparent contradiction might be linked to the fact that the high prevalence of multiple-partner fertility in the United States is partly explained by large shares of adolescent pregnancy and childbearing outside of unions (Thomson et al., 2020), rather than by childbearing in successive conjugal unions. This result is also in line with the weaker association we observed between the share of repartnered women and the share of cumulated fertility in second or subsequent unions in the United States. The lack of generous family and fertility policies in the United States could be a contributing factor to this result, as it has been shown that family policy measures appear to play a role in childbearing in second unions (Fernández Soto et al., 2020). This result should nonetheless be further confirmed with newer data, given that it was found only in the smallest of the United States (2007) datasets, but not in the larger one.

Analysing the association between union instability and fertility focusing only on ever- repartnered women allowed us to further disentangle the association between cumulated fertility and the amount of time spent in a first union rather than time spent in a second or subsequent union (net of age at first and second union formation and other confounders). This analysis revealed that, in most settings, time spent in a union *of any rank* is a positive contributing factor to cumulated fertility, with the notable exception of some Central and Eastern European countries. This finding is consistent with the historic evolution of fertility in these European regions, where childlessness is still not socially accepted but higher order parity transitions have been rare among women born since the 1940s birth cohorts (Zeman et al., 2018). In this context, time in union might matter less, as fertility goals might be sought soon after the start of the first union.

The positive association observed in most countries between the amount of time spent in a conjugal union and cumulated fertility by age 40 confirms previous findings by Thomson et al. (2012) for France, for a larger set of countries and regions. In this sense, our results are in line with much of the literature on multiple-partner fertility which suggests it is necessary to understand reproductive trajectories in contemporary societies as a continuum between successive partnerships. Our analysis reveals that despite differing patterns of union instability across regions, some of the key mechanisms in the relationship between union instability and fertility, namely the time in a union and the timing of union formation during the life course, act mostly similarly across national contexts and regions. Our findings suggest that the time spent in union during a woman's fertile years is a critical determinant of cumulated fertility achieved during that partnership succession, even net of age at first union formation and at repartnering. Age at union dissolution and early onset of conjugal life have been shown to be key elements in the likelihood of having a child in a new union (Beaujouan & Solaz, 2008; Kalmijn & Gelissen, 2007; Spijker et al., 2012). Our findings also add some nuance to the literature, suggesting that not only the timing of first and second or subsequent unions is critical, but also spending enough reproductive time in *any* of those unions so that fertility goals can be achieved during the reproductive ages.

Even though we found geographical patterns in the levels of union instability, the levels of repartnering, and in the importance of births in second or subsequent unions during childbearing ages, we did not find clear-cut geographical patterns in the relationship between union instability and fertility in the multivariate models linking the conjugal trajectory and cumulated fertility, once we estimated this relationship net of other confounders that vary greatly across regions (age at union formation and time spent in union among others). The fact that a positive association between repartnering and fertility is found in several countries despite their differing levels of union instability and overall fertility suggests that the experience of multiple partnerships might become a propelling factor for fertility -or at least an attenuating factor for decreasing fertility- in some sociocultural contexts. However, this can only have a visible global impact on fertility levels if repartnering becomes more prevalent following union dissolution: even a positive relationship between repartnering and cumulated fertility would have a negligible aggregate effect in regions where second union prevalence is very low such as in Southern, Central and Eastern Europe.

Finally, the main limitation of our study is that we were unable to incorporate factors like the presence, number, and age of children from previous unions for women and their partners (Beaujouan & Solaz, 2008; Griffith et al., 1985). Our data source does not contain information about the fertility trajectories of the respondent’s previous partners. In addition, our study examines cumulated fertility as the central variable of interest, and thus adding previous parity as a covariate would introduce endogeneity problems in the models.

Future work should focus on the relationship between union and fertility dynamics in a wider range of Latin American countries or sub-regions, representing diverging stages of the fertility transition.

## Data Availability

Data is publicly available upon request through the Generations and Gender website or from the authors.

## Appendix

**Table 1:** Descriptive statistics. Ever-partnered women aged 40 and older at the time of the survey. By regions, country and survey iteration

Regions	Country	Country abbreviations	Survey year	Num.of obs	Mean age at the survey	Has higher education (%)	Had child before 40 (%)	Conjugal status by age 40			Married in first union (%)	Mean age at first birth	Mean age at first union	Mean time spent in first union (years)	Mean age at first separation	Mean age at second union
								First union	Separated (no repartnered)	Repartnered						
Southern Europe	Spain	ESP 06	2006	4361	58.1	10.2	91.2	94.6	3.0	2.4	96.6	25.7	24.2	16.3	31.4	36.7
					(0.21)							(0.09)	(0.09)	(0.10)	(0.45)	(0.74)
	Spain	ESP 18	2018	6141	47.7	31.2	79.0	89.2	6.2	4.6	87.3	30.2	26.7	13.3	32.4	33.6
					(0.07)							(0.09)	(0.09)	(0.09)	(0.28)	(0.50)
	Italy	ITA	2003	2363	52.4	8.2	92.6	94.5	4.4	1.1	99.5	25.5	23.8	16.7	32.9	33.6
					(0.16)							(0.10)	(0.10)	(0.11)	(0.50)	(1.41)
Central Europe	Czech Republic	CZE	2005	1889	54.7	11.3	85.4	73.9	15.0	11.1	97.1	23.4	22.3	16.0	30.9	34.6
					(0.23)							(0.10)	(0.11)	(0.15)	(0.26)	(0.50)
	Estonia	EST	2004–2005	2282	55.9	36.3	94.8	62.3	14.7	23.0	92.7	23.9	22.5	13.9	29.3	32.1
					(0.23)							(0.09)	(0.08)	(0.14)	(0.20)	(0.29)
	Hungary	HUN	2004–2005	3086	55.4	14.4	93.3	71.0	13.7	15.3	97.0	23.0	21.2	16.4	29.8	32.4
					(0.18)							(0.08)	(0.07)	(0.13)	(0.22)	(0.35)
	Lithuania	LTU	2006	1679	54.8	23.7	91.7	74.2	17.9	7.9	96.3	24.9	23.6	14.8	31.5	34.9
					(0.25)							(0.11)	(0.12)	(0.15)	(0.27)	(0.59)
	Poland	POL	2010–2011	4699	57.0	15.3	94.4	84.5	10.3	5.2	98.0	23.5	22.5	16.8	30.8	34.5
					(0.14)							(0.06)	(0.06)	(0.08)	(0.22)	(0.45)
Eastern Europe	Bulgaria	BRG	2004	2471	55.8	21.9	93.8	91.1	5.1	3.8	97.6	23.0	21.3	18.6	29.6	31.9
					(0.22)							(0.10)	(0.09)	(0.11)	(0.43)	(0.89)
	Romania	ROU	2005	2736	56.2	8.3	90.0	89.3	6.0	4.7	97.5	23.5	21.7	18.1	29.8	32.7
					(0.20)							(0.09)	(0.08)	(0.11)	(0.36)	(0.69)
	Belarus	BLR	2017	1965	55.3	29.2	95.7	79.3	10.8	9.8	96.3	23.3	22.5	16.0	29.2	32.4
					(0.23)							(0.10)	(0.12)	(0.16)	(0.29)	(0.52)
	Georgia	GEO	2006	2217	53.9	25.2	95.5	92.8	5.3	1.9	94.8	24.2	23.1	17.0	29.4	31.0
					(0.22)							(0.11)	(0.11)	(0.12)	(0.52)	(0.97)
	Russia	RUS	2004	2766	53.1	44.8	95.5	67.6	13.4	19.0	94.1	23.7	22.4	14.4	28.6	31.6
					(0.19)							(0.09)	(0.09)	(0.15)	(0.20)	(0.30)
Western-Northern Europe	Austria	AUT	2008–2009	699	42.9	19.4	86.8	61.7	13.2	25.1	76.5	25.4	22.5	13.9	29.4	30.2
					(0.08)							(0.23)	(0.20)	(0.31)	(0.40)	(0.47)
	Germany	DEU	2008–2010	1304	41.5	38.7	82.6	62.3	9.9	27.8	85.5	28.4	24.0	11.9	28.2	30.3
					(0.03)							(0.22)	(0.18)	(0.24)	(0.30)	(0.34)
	Belgium	BEL	2008–2010	1756	55.5	32.1	83.1	68.3	7.8	23.8	84.0	25.8	22.1	14.5	27.5	30.0
					(0.24)							(0.12)	(0.12)	(0.17)	(0.30)	(0.39)
	France	FRA	2005	2373	55.3	20.6	91.4	76.7	9.3	14.0	87.7	24.7	22.4	16.0	30.2	33.9
					(0.24)							(0.11)	(0.09)	(0.14)	(0.26)	(0.40)
	UK	GBR	2005–2006	2042	55.0	45.9	88.8	72.2	9.3	18.6	90.5	25.8	22.8	14.7	28.9	32.5
					(0.29)							(0.14)	(0.12)	(0.19)	(0.31)	(0.45)
	Netherlands	NLD 03	2003	1965	50.3	19.5	87.5	76.8	9.7	13.5	91.5	26.1	22.6	15.8	30.2	32.5
					(0.16)							(0.13)	(0.10)	(0.16)	(0.33)	(0.43)
	Netherlands	NLD 13	2013	2879	55.9	28.1	86.6	69.9	10.6	19.5	83.2	26.9	23.1	14.3	29.4	32.5
					(0.20)							(0.11)	(0.09)	(0.13)	(0.22)	(0.32)
	Norway	NOR	2007–2008	3343	54.4	26.9	91.0	67.4	12.1	20.6	85.3	24.6	22.6	14.6	29.7	33.3
					(0.19)							(0.10)	(0.09)	(0.13)	(0.20)	(0.28)
	Sweden	SWE	2012–2013	2573	56.5	38.2	91.0	55.7	10.6	33.7	65.8	26.0	22.4	12.6	27.7	31.1
					(0.21)							(0.11)	(0.09)	(0.15)	(0.18)	(0.25)
North America	Canada	CAN 06	2006	5488	54.0	40.8	85.2	70.4	11.3	18.3	89.3	25.2	23.1	14.5	29.8	33.5
					(0.15)							(0.09)	(0.08)	(0.11)	(0.16)	(0.24)
	Canada	CAN 11	2011	5686	55.0	44.7	86.1	68.8	11.4	19.8	87.9	25.8	23.2	14.0	29.2	33.4
					(0.15)							(0.09)	(0.08)	(0.11)	(0.16)	(0.25)
	USA	USA 95	1995	1705	42.4	26.7	85.8	49.3	13.3	37.4	90.9	23.8	21.6	12.5	27.6	29.6
					(0.04)							(0.16)	(0.11)	(0.20)	(0.23)	(0.25)
	USA	USA 07	2006–2008	907	42.5	34.5	89.0	49.6	12.3	38.1	81.8	25.3	23.2	11.2	28.3	29.6
					(0.08)							(0.31)	(0.27)	(0.45)	(0.46)	(0.54)
Latin America	Colombia	COL	2015	6838	44.9	25.6	96.5	60.4	15.0	24.5	48.0	22.5	22.2	13.6	28.2	30.4
					(0.05)							(0.11)	(0.13)	(0.15)	(0.20)	(0.27)
	Mexico	MEX	2017	3982	46.7	13.9	96.7	76.2	11.9	11.8	81.7	22.4	21.5	16.4	28.8	31.5
					(0.09)							(0.12)	(0.13)	(0.16)	(0.29)	(0.41)
	Uruguay	URY	2008	678	50.3	24.0	92.2	77.6	10.0	12.4	71.3	24.8	24.1	14.5	30.3	34.8
					(0.25)							(0.22)	(0.27)	(0.30)	(0.54)	(0.82)

Standard errors in parentheses

*Source* Harmonised Histories (GGP), Family Situations Survey-Uruguay (2008), Demographic and Health Survey-Colombia (2015), Demographic Retrospective Survey-Mexico (2017)

**Table 2:** Cumulated fertility by rank of union to which births are attributable, on average (in number of children and as a percentage of total cumulated fertility) and share of repartnered women by childbearing trajectory: with births in first union only, in subsequent unions only, in first and subsequent unions, and without any births. Ever-partnered women aged 40 and older at the time of the survey. By regions, country and survey iteration

Regions	Country	Women ever in union								Repartnered women					
		Cumulated fertility, N				Cumulated fertility, %				Childbearing trajectory, %					
		Out of union	First union	Second + union	Total	Out of union	First union	Second + union	Total	First union	Second + union	First and second + union	Other	No children	Total
Southern Europe	ESP 06	0.09	2.15	0.03	2.27	4.0	94.7	1.3	100.0	24.9	20.3	20.0	16.2	18.5	100.0
		(0.01)	(0.02)	(0.00)	(0.02)										
	ESP 18	0.10	1.30	0.03	1.42	7.0	90.9	2.1	100.0	17.1	13.4	18.9	19.1	31.4	100.0
		(0.01)	(0.02)	(0.00)	(0.02)										
	ITA	0.04	1.79	0.01	1.84	2.2	97.3	0.5	100.0	32.4	19.5	24.7	13.3	10.2	100.0
		(0.00)	(0.02)	(0.00)	(0.02)										
Central Europe	CZE	0.15	1.47	0.06	1.69	8.9	87.5	3.6	100.0	29.7	31.6	7.2	18.7	12.9	100.0
		(0.01)	(0.02)	(0.01)	(0.02)										
	EST	0.13	1.66	0.20	1.99	6.5	83.4	10.1	100.0	27.1	35.2	10.1	21.0	6.7	100.0
		(0.01)	(0.02)	(0.01)	(0.02)										
	HUN	0.09	1.69	0.11	1.90	4.8	89.4	5.8	100.0	32.8	24.7	16.1	17.7	8.7	100.0
		(0.01)	(0.02)	(0.01)	(0.02)										
	LTU	0.11	1.59	0.05	1.75	6.3	90.9	2.9	100.0	42.0	25.6	11.0	13.9	7.5	100.0
		(0.01)	(0.02)	(0.01)	(0.02)										
	POL	0.10	2.08	0.04	2.22	4.5	93.7	1.8	100.0	26.4	28.9	8.5	26.4	9.8	100.0
		(0.01)	(0.02)	(0.00)	(0.02)										
Eastern Europe	BRG	0.05	1.68	0.04	1.77	2.8	94.9	2.3	100.0	20.6	32.0	18.1	16.6	12.6	100.0
		(0.01)	(0.02)	(0.01)	(0.02)										
	ROU	0.07	1.79	0.05	1.91	3.7	93.7	2.6	100.0	29.1	26.4	23.9	11.0	9.5	100.0
		(0.01)	(0.02)	(0.01)	(0.02)										
	BLR	0.15	1.61	0.08	1.83	8.2	87.5	4.3	100.0	26.5	37.0	6.9	26.1	3.6	100.0
		(0.01)	(0.02)	(0.01)	(0.02)										
	GEO	0.07	2.21	0.02	2.30	3.0	96.1	0.9	100.0	17.5	35.1	24.9	15.6	7.0	100.0
		(0.01)	(0.02)	(0.00)	(0.02)										
	RUS	0.12	1.55	0.16	1.83	6.6	84.7	8.7	100.0	24.8	34.4	14.4	19.6	6.7	100.0
		(0.01)	(0.02)	(0.01)	(0.02)										
Western-Northern Europe	AUT	0.16	1.42	0.18	1.76	9.1	80.7	10.2	100.0	25.2	13.6	30.5	11.9	18.7	100.0
		(0.03)	(0.05)	(0.02)	(0.05)										
	DEU	0.16	1.28	0.27	1.71	9.4	74.9	15.8	100.0	7.6	7.2	45.2	16.3	23.6	100.0
		(0.02)	(0.04)	(0.02)	(0.04)										
	BEL	0.08	1.47	0.27	1.81	4.4	80.8	14.8	100.0	21.1	7.6	45.8	9.1	16.3	100.0
		(0.01)	(0.03)	(0.02)	(0.03)										
	FRA	0.10	1.95	0.11	2.15	4.6	90.3	5.1	100.0	24.7	18.0	21.6	21.0	14.7	100.0
		(0.01)	(0.03)	(0.01)	(0.03)										
	GBR	0.10	1.80	0.18	2.08	4.8	86.5	8.7	100.0	25.3	16.1	27.4	16.1	15.1	100.0
		(0.01)	(0.03)	(0.02)	(0.03)										
	NLD 03	0.07	1.75	0.10	1.91	3.6	91.1	5.2	100.0	24.7	11.4	28.3	14.7	20.9	100.0
		(0.01)	(0.03)	(0.01)	(0.03)										
	NLD 13	0.07	1.65	0.19	1.90	3.7	86.4	9.9	100.0	17.5	8.4	40.7	11.6	21.9	100.0
		(0.01)	(0.02)	(0.01)	(0.02)										
	NOR	0.14	1.83	0.19	2.17	6.5	84.7	8.8	100.0	20.4	15.5	28.9	21.4	13.8	100.0
		(0.01)	(0.02)	(0.01)	(0.02)										
	SWE	0.12	1.53	0.41	2.06	5.8	74.3	19.9	100.0	17.3	14.8	45.7	11.1	11.1	100.0
		(0.01)	(0.02)	(0.02)	(0.02)										
North America	CAN 06	0.13	1.78	0.14	2.04	6.3	86.8	6.8	100.0	25.2	10.2	23.4	20.9	20.3	100.0
		(0.01)	(0.02)	(0.01)	(0.02)										
	CAN 11	0.13	1.69	0.16	1.97	6.6	85.4	8.1	100.0	22.6	11.3	27.4	20.6	18.2	100.0
		(0.01)	(0.02)	(0.01)	(0.02)										
	USA 95	0.23	1.55	0.28	2.07	11.2	75.2	13.6	100.0	22.8	14.1	20.5	26.8	15.8	100.0
		(0.02)	(0.03)	(0.02)	(0.04)										
	USA 07	0.36	1.58	0.36	2.29	15.7	68.7	15.7	100.0	12.8	8.5	29.6	34.5	14.6	100.0
		(0.04)	(0.09)	(0.06)	(0.08)										
Latin America	COL	0.50	2.05	0.31	2.85	17.5	71.7	10.8	100.0	16.5	25.2	3.8	51.4	3.1	100.0
		(0.02)	(0.03)	(0.02)	(0.03)										
	MEX	0.30	2.50	0.14	2.94	10.2	85.0	4.8	100.0	16.7	22.0	5.8	53.8	1.8	100.0
		(0.01)	(0.04)	(0.01)	(0.04)										
	URY	0.33	1.85	0.15	2.32	14.2	79.4	6.4	100.0	14.5	25.3	21.1	30.5	8.6	100.0
		(0.03)	(0.06)	(0.02)	(0.06)										

*Source* Harmonised Histories (GGP), Family Situations Survey-Uruguay (2008), Demographic and Health Survey-Colombia (2015), Demographic Retrospective Survey-Mexico (2017)

Standard errors in parentheses

**Table 3:** Model 1: Exponentiated coefficients of poisson regression models for ever-partnered women. Ever-partnered women aged 40 and older at the time of the survey. By regions, country and survey iteration

Number of children at age 40	Southern Europe			Central Europe					Eastern Europe				
	ESP06	ESP18	ITA	CZE	EST	HUN	LTU	POL	BRG	ROU	BLR	GEO	RUS
Ever experienced separation	0.856**	0.841***	0.768***	0.947	0.903***	0.910***	0.852***	0.791***	0.846***	0.866**	0.939*	0.693***	0.880***
(ref. In first union)	(0.05)	(0.04)	(0.04)	(0.03)	(0.02)	(0.02)	(0.03)	(0.02)	(0.03)	(0.04)	(0.03)	(0.04)	(0.02)
No union before age 25	0.742***	0.787***	0.769***	0.770***	0.811***	0.741***	0.810***	0.759***	0.781***	0.694***	0.789***	0.791***	0.720***
(ref. First union before age 25)	(0.02)	(0.02)	(0.02)	(0.03)	(0.03)	(0.03)	(0.03)	(0.02)	(0.03)	(0.03)	(0.03)	(0.02)	(0.02)
Higher educational attainment	0.962	1.007	0.981	0.890**	0.892***	0.946*	0.896***	0.799***	0.853***	0.735***	0.884***	0.870***	0.880***
(ref.: No)	(0.04)	(0.02)	(0.04)	(0.04)	(0.02)	(0.02)	(0.03)	(0.02)	(0.02)	(0.03)	(0.02)	(0.02)	(0.02)
Age	1.010***	0.986***	1.006***	0.994***	0.993***	0.993***	0.997	1.000	0.994***	0.999	1.003*	1.003**	0.998*
	(0.00)	(0.00)	(0.00)	(0.00)	(0.00)	(0.00)	(0.00)	(0.00)	(0.00)	(0.00)	(0.00)	(0.00)	(0.00)
Marriage	1.769***	1.469***	3.119***	1.761***	0.981	1.073	1.044	1.281**	0.902	0.892	1.135	1.284***	1.186**
(ref.: Consensual union)	(0.18)	(0.07)	(0.95)	(0.21)	(0.06)	(0.14)	(0.11)	(0.10)	(0.07)	(0.09)	(0.10)	(0.08)	(0.06)
Constant	0.809	2.373***	0.484*	1.407*	3.401***	2.808***	2.169***	1.910***	2.954***	2.477***	1.496***	1.736***	2.055***
	(0.09)	(0.28)	(0.15)	(0.20)	(0.28)	(0.38)	(0.29)	(0.18)	(0.28)	(0.31)	(0.16)	(0.14)	(0.16)
Observations	3842	6167	2363	1858	2282	3086	1678	4693	2440	2736	1960	2216	2628

Number of children at age 40	Western-Northern Europe									North America				Latin America		
	AUT	DEU	BEL	FRA	GBR	NLD03	NLD13	NOR	SWE	CAN06	CAN11	USA95	USA07	COL	MEX	URY
Ever experienced separation	0.833**	0.817***	0.951	0.910**	1.014	0.897**	0.855***	0.855***	0.949*	0.888***	0.857***	0.897**	0.809**	0.966	0.962	0.891
(ref. In first union)	(0.05)	(0.05)	(0.04)	(0.03)	(0.04)	(0.04)	(0.03)	(0.02)	(0.02)	(0.02)	(0.02)	(0.03)	(0.06)	(0.02)	(0.02)	(0.08)
No union before age 25	0.784***	0.878**	0.796***	0.748***	0.784***	0.807***	0.801***	0.758***	0.757***	0.694***	0.682***	0.624***	0.818**	0.692***	0.647***	0.699***
(ref. Frst union before age 25)	(0.05)	(0.04)	(0.04)	(0.03)	(0.04)	(0.03)	(0.03)	(0.02)	(0.02)	(0.02)	(0.02)	(0.04)	(0.06)	(0.02)	(0.02)	(0.04)
Higher educational attainment	0.855**	0.942	1.061	0.959	0.906**	0.980	0.953	1.000	1.009	0.972	0.949**	0.849***	0.858*	0.724***	0.773***	0.857***
(ref.: No)	(0.05)	(0.05)	(0.04)	(0.03)	(0.03)	(0.04)	(0.03)	(0.02)	(0.02)	(0.02)	(0.02)	(0.03)	(0.06)	(0.02)	(0.02)	(0.04)
Age	0.991	0.989	0.996*	1.002	1.001	1.003	1.001	1.000	0.997**	1.010***	1.005***	0.999	0.967	1.012***	1.014***	1.001
	(0.02)	(0.03)	(0.00)	(0.00)	(0.00)	(0.00)	(0.00)	(0.00)	(0.00)	(0.00)	(0.00)	(0.01)	(0.02)	(0.00)	(0.00)	(0.00)
Marriage	1.439***	2.003***	1.126*	1.442***	1.408***	1.704***	1.494***	1.290***	1.272***	1.487***	1.408***	1.397***	1.032	0.896***	0.966	0.972
(ref.: Consensual union)	(0.12)	(0.28)	(0.06)	(0.08)	(0.12)	(0.19)	(0.08)	(0.05)	(0.04)	(0.07)	(0.06)	(0.14)	(0.11)	(0.02)	(0.03)	(0.08)
Constant	2.230	1.730	2.129***	1.486***	1.609***	1.061	1.417***	1.957***	2.252***	0.953	1.276***	1.806	11.158**	2.041***	1.754***	2.679***
	(1.61)	(2.17)	(0.26)	(0.13)	(0.20)	(0.14)	(0.11)	(0.14)	(0.13)	(0.07)	(0.09)	(0.86)	(10.23)	(0.30)	(0.22)	(0.66)
Observations	699	1303	1753	2373	1729	1961	2798	3332	2568	5402	5631	1705	907	6839	3809	665

Exponentiated coefficients; Standard errors in parentheses

*Source* Harmonised Histories (GGP), Family Situations Survey-Uruguay (2008), Demographic and Health Survey-Colombia (2015), Demographic Retrospective Survey-Mexico (2017)

**p* < 0.05, ***p* < 0.01, ****p* < 0.001

**Table 4:** Model 2: Exponentiated coefficients of Poisson regression models for ever-partnered women. Ever-partnered women aged 40 and older at the time of the survey. By regions, country and survey iteration

Number of children at age 40	Southern Europe			Central Europe					Eastern Europe				
	ESP06	ESP18	ITA	CZE	EST	HUN	LTU	POL	BRG	ROU	BLR	GEO	RUS
Ever experienced separation	0.779***	0.782***	0.755***	0.814***	0.764***	0.843***	0.791***	0.735***	0.724***	0.764***	0.819***	0.626***	0.779***
	(0.06)	(0.06)	(0.04)	(0.04)	(0.03)	(0.03)	(0.03)	(0.02)	(0.03)	(0.05)	(0.03)	(0.04)	(0.03)
Repartnered	0.954	0.913	0.817	1.129**	0.992	0.968	0.989	0.898**	1.010	0.997	1.073	0.886	0.951
(ref. In first union)	(0.08)	(0.06)	(0.10)	(0.05)	(0.03)	(0.03)	(0.06)	(0.04)	(0.06)	(0.07)	(0.04)	(0.09)	(0.03)
No union before age 25	0.743***	0.788***	0.769***	0.783***	0.827***	0.749***	0.819***	0.762***	0.784***	0.697***	0.798***	0.793***	0.727***
(ref. First union before age 25)	(0.02)	(0.02)	(0.02)	(0.03)	(0.03)	(0.03)	(0.03)	(0.02)	(0.03)	(0.03)	(0.03)	(0.02)	(0.02)
Higher educational attainment	0.961	1.006	0.982	0.887**	0.896***	0.946*	0.900***	0.799***	0.855***	0.738***	0.887***	0.869***	0.878***
(ref.: No)	(0.04)	(0.02)	(0.04)	(0.04)	(0.02)	(0.02)	(0.03)	(0.02)	(0.02)	(0.03)	(0.02)	(0.02)	(0.02)
Age	1.010***	0.986***	1.006***	0.994***	0.993***	0.993***	0.997	1.000	0.994***	0.999	1.003*	1.003**	0.998*
	1.780***	1.464***	3.120***	1.825***	0.982	1.073	1.051	1.290**	0.923	0.915	1.160	1.301***	1.197***
Marriage	(0.18)	(0.07)	(0.95)	(0.23)	(0.06)	(0.14)	(0.11)	(0.10)	(0.07)	(0.09)	(0.10)	(0.08)	(0.06)
(ref.: Consensual union)	0.802*	2.362***	0.484*	1.375*	3.418***	2.815***	2.154***	1.896***	2.884***	2.417***	1.451***	1.717***	2.018***
Constant	(0.09)	(0.27)	(0.15)	(0.20)	(0.28)	(0.38)	(0.28)	(0.17)	(0.27)	(0.30)	(0.15)	(0.14)	(0.16)
Observations	3842	6167	2363	1858	2282	3086	1678	4693	2440	2736	1960	2216	2628

Number of children at age 40	Western-Northern Europe									North America				Latin America		
	AUT	DEU	BEL	FRA	GBR	NLD03	NLD13	NOR	SWE	CAN06	CAN11	USA95	USA07	COL	MEX	URY
Ever experienced separation	0.747*	0.778**	0.902	0.855***	0.858**	0.850*	0.776***	0.782***	0.843***	0.868***	0.817***	0.902	0.957	0.825***	0.838***	0.863
	(0.10)	(0.08)	(0.06)	(0.04)	(0.05)	(0.06)	(0.04)	(0.03)	(0.03)	(0.03)	(0.03)	(0.05)	(0.09)	(0.02)	(0.02)	(0.10)
Repartnered	0.894	0.828**	0.979	0.946	1.110*	0.932	0.915*	0.910**	1.004	0.902***	0.884***	0.895**	0.746**	1.057*	1.089**	0.913
(ref. In first union)	(0.06)	(0.05)	(0.05)	(0.04)	(0.05)	(0.05)	(0.04)	(0.03)	(0.03)	(0.02)	(0.02)	(0.03)	(0.07)	(0.03)	(0.03)	(0.09)
No union before age 25	0.796***	0.879**	0.800***	0.751***	0.794***	0.811***	0.809***	0.764***	0.767***	0.695***	0.685***	0.623***	0.802**	0.704***	0.653***	0.700***
(ref. First union before age 25)	(0.05)	(0.04)	(0.04)	(0.03)	(0.04)	(0.03)	(0.03)	(0.02)	(0.02)	(0.02)	(0.02)	(0.04)	(0.06)	(0.02)	(0.02)	(0.04)
Higher educational attainment	0.852**	0.941	1.060	0.959	0.908**	0.979	0.952	1.000	1.011	0.972	0.950**	0.849***	0.859*	0.728***	0.776***	0.857***
(ref.: No)	(0.05)	(0.05)	(0.04)	(0.03)	(0.03)	(0.04)	(0.03)	(0.02)	(0.02)	(0.02)	(0.02)	(0.03)	(0.06)	(0.02)	(0.03)	(0.04)
Age	0.990	0.988	0.996	1.002	1.001	1.003	1.001	1.000	0.997**	1.010***	1.005***	0.999	0.969	1.013***	1.014***	1.001
	1.478***	1.984***	1.150*	1.443***	1.461***	1.709***	1.536***	1.324***	1.309***	1.494***	1.418***	1.396***	0.989	0.911***	0.983	0.973
Marriage	(0.12)	(0.28)	(0.07)	(0.08)	(0.12)	(0.19)	(0.08)	(0.05)	(0.04)	(0.07)	(0.07)	(0.14)	(0.11)	(0.02)	(0.03)	(0.08)
(ref.: Consensual union)	2.254	1.755	2.079***	1.479***	1.528***	1.061	1.362***	1.887***	2.161***	0.944	1.254**	1.809	10.797**	1.931***	1.722***	2.655***
Constant	(1.63)	(2.20)	(0.26)	(0.13)	(0.19)	(0.14)	(0.11)	(0.13)	(0.12)	(0.07)	(0.09)	(0.86)	(9.57)	(0.28)	(0.22)	(0.66)
Observations	699	1303	1753	2373	1729	1961	2798	3332	2568	5402	5631	1705	907	6839	3809	665

*Source* Harmonised Histories (GGP), Family Situations Survey-Uruguay (2008), Demographic and Health Survey-Colombia (2015), Demographic Retrospective Survey-Mexico (2017)

Exponentiated coefficients; Standard errors in parentheses

^*^ *p* < 0.05, ** *p* < 0.01, *** *p* < 0.001

**Table 5:** Model 3: Exponentiated coefficients of Poisson regression models for ever-partnered women. Ever-partnered women aged 40 and older at the time of the survey. By regions, country and survey iteration

Number of children at age 40	Southern Europe			Central Europe					Eastern Europe				
	ESP06	ESP18	ITA	CZE	EST	HUN	LTU	POL	BRG	ROU	BLR	GEO	RUS
Ever experienced separation	1.048	0.966	1.065	0.985	0.922	1.125*	1.028	1.030	1.021	1.254**	0.986	0.992	0.961
	(0.08)	(0.07)	(0.06)	(0.06)	(0.05)	(0.07)	(0.05)	(0.04)	(0.06)	(0.09)	(0.04)	(0.06)	(0.04)
Repartnered	1.098	0.951	0.973	1.227***	1.067*	1.101**	1.119	1.056	1.107	1.146*	1.169***	1.056	1.036
(ref. In first union)	(0.09)	(0.07)	(0.13)	(0.06)	(0.03)	(0.04)	(0.07)	(0.04)	(0.07)	(0.08)	(0.05)	(0.11)	(0.03)
Total time spent in union by age 40	1.036***	1.036***	1.048***	1.021***	1.021***	1.033***	1.033***	1.040***	1.035***	1.054***	1.019***	1.048***	1.022***
	(0.00)	(0.00)	(0.00)	(0.00)	(0.00)	(0.00)	(0.00)	(0.00)	(0.00)	(0.00)	(0.00)	(0.00)	(0.00)
No union before age 25	0.957	1.034	1.084*	0.927	0.942	0.962	1.030	1.013	1.071	1.077	0.941	1.148***	0.862***
(ref. First union before age 25)	(0.03)	(0.04)	(0.04)	(0.05)	(0.04)	(0.05)	(0.04)	(0.03)	(0.05)	(0.06)	(0.04)	(0.04)	(0.03)
Higher educational attainment	1.000	1.033	1.014	0.906*	0.906***	0.984	0.902***	0.830***	0.905***	0.808***	0.902***	0.913***	0.887***
(ref.: No)	(0.04)	(0.02)	(0.04)	(0.04)	(0.02)	(0.03)	(0.03)	(0.02)	(0.02)	(0.03)	(0.02)	(0.02)	(0.02)
Age	1.011***	0.987***	1.005***	0.995***	0.994***	0.994***	0.998	1.001	0.994***	1.000	1.004**	1.004***	0.999
	(0.00)	(0.00)	(0.00)	(0.00)	(0.00)	(0.00)	(0.00)	(0.00)	(0.00)	(0.00)	(0.00)	(0.00)	(0.00)
Marriage	1.818***	1.316***	2.743**	1.710***	0.952	1.037	1.003	1.235**	0.906	0.891	1.109	1.195**	1.157**
(ref.: Consensual union)	(0.17)	(0.06)	(0.88)	(0.21)	(0.06)	(0.13)	(0.10)	(0.09)	(0.07)	(0.09)	(0.10)	(0.07)	(0.06)
Constant	0.366***	1.254	0.218***	0.940	2.197***	1.429*	1.197	0.895	1.354*	0.793	0.993	0.671***	1.258*
	(0.05)	(0.17)	(0.07)	(0.16)	(0.26)	(0.23)	(0.18)	(0.10)	(0.17)	(0.12)	(0.11)	(0.07)	(0.13)
Observations	3842	6167	2363	1858	2282	3086	1678	4693	2440	2736	1960	2216	2628

Number of children at age 40	Western-Northern Europe									North America				Latin America		
	AUT	DEU	BEL	FRA	GBR-	NLD03	NLD13	NOR	SWE	CAN06	CAN11	USA95	USA07	COL	MEX	URY
Ever experienced separation	0.986	0.986	1.058	1.131*	1.179*	0.974	0.997	0.997	1.045	1.178***	1.079	1.132	1.302*	1.047	1.141***	0.973
	(0.12)	(0.10)	(0.07)	(0.06)	(0.08)	(0.07)	(0.05)	(0.04)	(0.04)	(0.04)	(0.05)	(0.07)	(0.14)	(0.03)	(0.04)	(0.12)
Repartnered	1.010	0.945	0.994	1.057	1.243***	0.992	1.022	1.020	1.071*	1.018	0.991	1.016	0.814*	1.178***	1.272***	0.972
(ref. In first union)	(0.06)	(0.06)	(0.06)	(0.05)	(0.06)	(0.05)	(0.04)	(0.03)	(0.03)	(0.03)	(0.03)	(0.04)	(0.07)	(0.03)	(0.04)	(0.10)
Total time spent in union by age 40	1.037***	1.039***	1.022***	1.034***	1.039***	1.019***	1.033***	1.032***	1.025***	1.037***	1.030***	1.032***	1.044***	1.030***	1.037***	1.015**
	(0.01)	(0.01)	(0.00)	(0.00)	(0.01)	(0.01)	(0.00)	(0.00)	(0.00)	(0.00)	(0.00)	(0.00)	(0.01)	(0.00)	(0.00)	(0.01)
No union before age 25	1.069	1.147*	0.952	0.959	1.054	0.925	1.015	0.970	0.930*	0.932*	0.865***	0.799**	1.085	0.913**	0.916*	0.824**
(ref. First union before age 25)	(0.11)	(0.08)	(0.06)	(0.05)	(0.06)	(0.05)	(0.04)	(0.03)	(0.03)	(0.03)	(0.03)	(0.06)	(0.10)	(0.03)	(0.03)	(0.06)
Higher educational attainment	0.872*	0.961	1.082*	0.999	0.943	0.992	0.980	1.033	1.030	1.003	0.979	0.887**	0.883	0.761***	0.829***	0.866**
(ref.: No)	(0.05)	(0.05)	(0.04)	(0.03)	(0.03)	(0.04)	(0.03)	(0.02)	(0.02)	(0.02)	(0.02)	(0.04)	(0.06)	(0.02)	(0.03)	(0.04)
Age	0.987	0.977	0.997	1.004*	1.002	1.004	1.002*	1.001	0.998	1.010***	1.006***	0.996	0.977	1.014***	1.015***	1.002
	(0.02)	(0.03)	(0.00)	(0.00)	(0.00)	(0.00)	(0.00)	(0.00)	(0.00)	(0.00)	(0.00)	(0.01)	(0.02)	(0.00)	(0.00)	(0.00)
Marriage	1.421***	1.727***	1.123	1.377***	1.351***	1.630***	1.466***	1.258***	1.252***	1.408***	1.358***	1.258*	0.931	0.903***	0.961	0.954
(ref.: Consensual union)	(0.11)	(0.23)	(0.07)	(0.07)	(0.11)	(0.18)	(0.08)	(0.05)	(0.04)	(0.07)	(0.06)	(0.12)	(0.09)	(0.02)	(0.03)	(0.08)
Constant	1.302	1.574	1.326	0.732*	0.728*	0.737	0.714**	1.002	1.286**	0.470***	0.692***	1.192	3.531	0.980	0.759*	1.867*
	(0.93)	(1.95)	(0.21)	(0.10)	(0.12)	(0.12)	(0.08)	(0.10)	(0.11)	(0.04)	(0.06)	(0.56)	(3.07)	(0.15)	(0.10)	(0.51)
Observations	699	1303	1753	2373	1729	1961	2798	3332	2568	5402	5631	1705	907	6839	3809	665

Source: Harmonised Histories (GGP), Family Situations Survey-Uruguay (2008), Demographic and Health Survey-Colombia (2015), Demographic Retrospective Survey-Mexico (2017)

Exponentiated coefficients; Standard errors in parentheses

^*^ *p* < 0.05, ** *p* < 0.01, *** *p* < 0.001

**Table 6:** Model 4: Exponentiated coefficients of Poisson regression models for ever-repartnered women by age 40. Ever-repartnered women aged 40 and older at the time of the survey. By regions, country and survey iteration

	Southern Europe		Central Europe					Eastern Europe			
Number of children at age 40	ESP06	ESP18	CZE	EST	HUN	LTU	POL	BRG	ROU	BLR	RUS
Time in union 1	1.071**	1.005	1.019	1.023**	1.033***	1.030*	1.018	1.008	1.055**	1.013	1.013
	(0.02)	(0.01)	(0.01)	(0.01)	(0.01)	(0.01)	(0.01)	(0.02)	(0.02)	(0.01)	(0.01)
Time in union 2 +	1.048**	1.027	1.018	1.002	1.022***	1.000	1.031***	1.008	1.074**	1.019*	1.016**
	(0.02)	(0.02)	(0.01)	(0.01)	(0.01)	(0.02)	(0.01)	(0.01)	(0.02)	(0.01)	(0.01)
First union after age 25	1.343	0.746*	0.856	0.809	0.624	0.744	0.812	0.959	1.214	0.689*	0.748**
(ref. First union before age 25)	(0.25)	(0.11)	(0.21)	(0.09)	(0.17)	(0.18)	(0.14)	(0.32)	(0.43)	(0.10)	(0.08)
Repartnering after age 30	0.991	0.915	0.894	0.850*	0.966	0.843	1.020	0.742	1.232	1.070	0.955
(ref. Repartnering before age 30)	(0.22)	(0.14)	(0.12)	(0.06)	(0.08)	(0.12)	(0.11)	(0.12)	(0.28)	(0.10)	(0.07)
Higher educational attainment	0.764	0.652*	0.723**	0.852**	0.856*	0.842	0.708***	0.734	0.751	0.748***	0.840***
(ref.: No)	(0.13)	(0.13)	(0.08)	(0.05)	(0.07)	(0.13)	(0.07)	(0.12)	(0.15)	(0.06)	(0.04)
Age	1.007	0.989	0.989*	0.996	0.992*	0.996	1.006	0.991	0.993	1.005	0.998
	(0.02)	(0.02)	(0.00)	(0.00)	(0.00)	(0.01)	(0.00)	(0.01)	(0.01)	(0.00)	(0.00)
Marriage	1.928*	1.203	2.759***	0.807**	0.906	0.759	1.138	0.778	1.140	0.959	1.087
(ref.: Consensual union)	(0.51)	(0.19)	(0.69)	(0.07)	(0.13)	(0.15)	(0.16)	(0.13)	(0.19)	(0.17)	(0.09)
Constant	0.342	2.057	1.095	3.111***	2.233***	2.731*	0.951	3.891***	0.764	1.272	1.709**
	(0.28)	(1.50)	(0.41)	(0.57)	(0.54)	(1.20)	(0.29)	(1.59)	(0.37)	(0.25)	(0.32)
Observations	101	254	207	525	445	136	246	103	131	222	545

	Western-Northern Europe									North America				Latin America	
Number of children at age 40	AUT	DEU	BEL	FRA	GBR	NLD03	NLD13	NOR	SWE	CAN06	CAN11	USA95	USA07	COL	MEX
Time in union 1	1.049***	1.059***	1.021	1.034***	1.037***	1.022	1.022*	1.039***	1.026***	1.027***	1.025***	1.038***	1.054**	1.021***	1.024***
	(0.01)	(0.02)	(0.01)	(0.01)	(0.01)	(0.01)	(0.01)	(0.01)	(0.01)	(0.01)	(0.01)	(0.01)	(0.02)	(0.00)	(0.01)
Time in union 2 +	1.072***	1.055**	1.020**	1.029**	1.030**	0.988	1.031***	1.024**	1.027***	1.027***	1.014**	1.018*	1.034***	1.015***	1.014**
	(0.02)	(0.02)	(0.01)	(0.01)	(0.01)	(0.01)	(0.01)	(0.01)	(0.00)	(0.01)	(0.00)	(0.01)	(0.01)	(0.00)	(0.00)
First union after age 25	0.991	1.375**	1.133	0.861	1.019	0.527	0.835	0.929	0.792*	0.700**	0.726**	0.765	1.112	0.766***	0.808*
(ref. First union before age 25)	(0.20)	(0.17)	(0.17)	(0.18)	(0.19)	(0.18)	(0.12)	(0.11)	(0.09)	(0.08)	(0.07)	(0.14)	(0.23)	(0.06)	(0.08)
Repartnering after age 30	1.167	1.040	0.825	0.862	0.966	0.790	0.983	0.881	0.966	0.881	0.854**	0.824*	0.863	0.814***	0.878*
(ref. Repartnering before age 30)	(0.20)	(0.15)	(0.11)	(0.09)	(0.12)	(0.11)	(0.09)	(0.07)	(0.06)	(0.06)	(0.05)	(0.07)	(0.12)	(0.04)	(0.05)
Higher educational attainment	0.754*	0.859	1.026	0.795*	0.959	0.931	0.826*	1.033	0.957	0.837***	0.893*	0.771**	0.855	0.756***	0.751***
(ref.: No)	(0.11)	(0.09)	(0.07)	(0.07)	(0.08)	(0.11)	(0.07)	(0.05)	(0.04)	(0.04)	(0.04)	(0.06)	(0.12)	(0.05)	(0.06)
Age	0.975	0.924	1.004	0.992	1.013**	1.007	1.000	0.997	0.996*	1.007*	1.002	1.034	0.933**	1.012	1.001
	(0.03)	(0.05)	(0.00)	(0.00)	(0.01)	(0.01)	(0.00)	(0.00)	(0.00)	(0.00)	(0.00)	(0.02)	(0.02)	(0.01)	(0.01)
Marriage	1.356**	1.766***	1.146	1.400***	1.139	1.205	1.343***	1.160*	1.215***	1.234**	1.190**	1.145	0.996	0.879*	0.844**
(ref.: Consensual union)	(0.16)	(0.27)	(0.12)	(0.14)	(0.12)	(0.17)	(0.11)	(0.08)	(0.06)	(0.08)	(0.07)	(0.11)	(0.08)	(0.05)	(0.04)
Constant	1.411	11.157	0.990	1.640	0.628	1.165	0.986	1.400	1.616***	0.827	1.239	0.329	20.946**	1.695	2.922***
	(2.16)	(26.50)	(0.27)	(0.42)	(0.21)	(0.53)	(0.26)	(0.32)	(0.23)	(0.15)	(0.20)	(0.25)	(22.50)	(0.49)	(0.84)
Observations	178	375	418	361	380	240	498	674	866	1102	1208	626	383	1848	559

Exponentiated coefficients; Standard errors in parentheses

*Source* Harmonised Histories (GGP), Family Situations Survey-Uruguay (2008), Demographic and Health Survey-Colombia (2015), Demographic Retrospective Survey-Mexico (2017)

^*^*p* < 0.05, ** *p* < 0.01, *** *p* < 0.001

## Footnotes

1 Estimated Total Fertility Rates in 2020 for these three Latin American countries are similar to those in some European countries in the same time period: Uruguay’s TFR of 1.48 is very similar to the European average (1.50) and equivalent to that of Norway (1.48), while fertility levels in Colombia (1.74) and Mexico (1.91) are comparable to those of the countries with highest fertility in Europe: for instance Georgia (1.98), France (1.83), Romania (1.80) and Iceland (1.72) (Cepalstat, 2023; Eurostat, 2023).

2 The national datasets are the Demographic and Health Survey (2015) for Colombia, the Family Situations Survey—*Encuesta de Situaciones Familiares-* (2008) for Uruguay and the Demographic Retrospective Survey—*Encuesta Demográfica Retrospectiva*- (2017) for Mexico.

3 We eliminated GGP Germany 2005 from the analysis based on the results of Kreyenfeld et al. (2011).

4 From the Harmonised Histories dataset, we selected countries where the age composition of the sample would allow for the age selection needed for our analysis. These countries are: Austria (Gender and Generation Survey (GGS) 2008-09), Belarus (GGS 2017), Belgium (GGS 2008-09), Bulgaria (GGS 2004), Canada (GSS 2006 and 2011), Czech Republic (GGS 2005), Estonia (GGS 2004-05), France (GGS 2005), Georgia (GGS 2006), Germany (Pairfam 2009), Hungary (GGS 2004-05), Italy (GGS 2003), Lithuania (GGS 2006), Netherlands (Fertility and Family Survey 2003 and 2013), Norway (GGS 2007-08), Poland (GGS 2010-11), Romania (GGS 2005), Russia (GGS 2004), Sweden (GGS 2012-13), the United Kingdom (British Household Panel Survey 2005-06) and the United States (National Survey of Family Growth 1995 and 2007).

5 We selected women aged 40 and over on the understanding that most family and fertility transitions occur before these ages. Additionally, some surveys included in the analysis only consider women of reproductive age (15-49 years). Some countries in the Harmonised Histories were dropped from the analysis due to an insufficient sample size meeting these criteria.

6 Widows were excluded from the analysis.

7 The category “other” refers mainly to births outside of union and includes the following situations: all births outside of union; births in first union and outside of union; births in second or subsequent union and outside of union; births in first and second or subsequent union and outside of union.

8 This decomposition is inspired from other decomposition techniques used in previous research such as: Laplante and Fostik (2015), Fernández Soto and Laplante (2020), and Fernández Soto et al., (2020).

9 We chose to use a continuous specification for this variable in order to be able to include countries with a low number of observations in the analysis, since a categorical specification would hinder the degrees of freedom of the models.

10 We use a continuous specification of time for these two variables as well.

11 See Table 2 in the appendix for more details of these values.

12 For the sake of clarity of the results, we only show the combinations of first and second union fertility. The values for the rest of the combinations are shown in the appendix.

13 See Table 2 in the appendix for more details of these values.

14 Table 3 in the Appendix shows the exponentiated coefficients for each country and survey year.

15 Table 4 in the appendix shows the exponentiated coefficients for each country and survey year.

16 Table 5 in the appendix shows the exponentiated coefficients for each country and survey year.

17 These models could not be conducted for Italy, Georgia, and Uruguay, due to the few observations in the subsample of repartnered women in these countries (see Table 6 in Appendix).

## Code Availability

Stata code is available upon request.
